# Synergistic effect of Pladienolide B and cisplatin: enhancing autophagy in hepatoma cells through the AMPK/mTOR/ULK1 pathway

**DOI:** 10.1038/s41420-026-03144-5

**Published:** 2026-05-08

**Authors:** Wei Xiao, Lei Yang, Ze Li, Wujie Wang, Zhaojian Liu, Bin Liu, Junchao Qin, Yuliang Li

**Affiliations:** 1 School of Clinical Medicine, Hebei University of Engineering, Handan, China; 2 Department of Interventional Medicine and Minimally Invasive Oncology, The Second Qilu Hospital of Shandong University, Jinan, China; 3 Institute of Interventional Oncology, Shandong University, Jinan, China; 4 Ministry of Education, Department of Cell Biology, School of Basic Medical Sciences, Shandong University, Jinan, China

**Keywords:** Chemotherapy, Cell biology

## Abstract

Alternative splicing (AS) is a key driver of development and a major contributor to species diversity. Accumulating evidence indicates its high activity in various cancers. Here, we identified the spliceosome component SF3B1 as a key regulator of cell fate in hepatocellular carcinoma (HCC), with its expression elevated in HCC tissues/cells versus adjacent non-tumor tissues. Using SF3B1 inhibitor Pladienolide B (Pla B), we found that it suppresses HCC cells' proliferation and induces apoptosis. RNA-Seq revealed Pla B modulates AS events in HCC cells; KEGG analysis indicated it affects the AMPK-mTOR pathway to activate autophagy. In vivo xenograft experiments further demonstrated that the combined treatment of Pla B and cisplatin achieved a more potent inhibitory effect on tumor growth compared to either monotherapy. This combinatorial strategy not only reduced tumor cell proliferation and promoted apoptosis but also enhanced autophagy. Collectively, our findings highlight the potential of combining Pla B with cisplatin as a novel and promising therapeutic approach for the treatment of HCC.

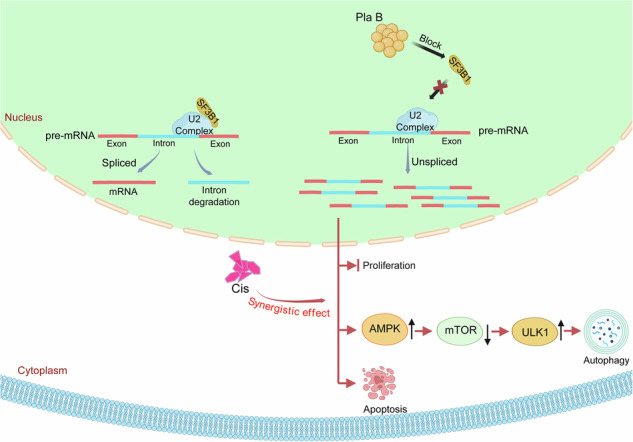

## Introduction

Primary liver cancer is the sixth most common cancer globally and the third leading cause of cancer-related deaths [1]. Projections indicate its incidence will rise by 55.0% from 2020 to 2040, with an estimated 1.4 million new cases and 1.3 million deaths expected in 2040 [2]. HCC accounts for 75–86% of primary liver cancer cases and ranks second in mortality among men [1, 3]. Risk factors for HCC include demographic variables (age, sex, ethnicity), underlying liver disease characteristics (fibrosis stage, inflammatory activity, treatment status), metabolic factors (diabetes, obesity), and lifestyle habits (alcohol consumption, smoking) [4]. For established HCC, potential interventions include surgery (liver resection, transplantation) [5], liver transplantation [6, 7], ablation [8, 9], intra-arterial therapies, radiotherapy [10, 11], and systemic therapy for advanced patients. Despite emerging systemic treatments, survival rates for advanced liver cancer remain poor [12]. The complex pathogenesis of HCC has motivated the exploration of various targeted therapies. An accurate grasp of its molecular mechanisms is crucial for developing effective treatments. Nevertheless, the efficacy of existing first- and second-line regimens is frequently hampered by serious drug resistance and side effects. Therefore, optimizing HCC treatment strategies represents a pressing global challenge.

AS is a vital RNA processing mechanism that generates diverse mRNA/protein subtypes from a single mRNA transcript, thereby expanding the diversity and complexity of the transcriptome and proteome [13, 14]. Genetic alterations and/or abnormal expression of spliceosome components can induce aberrant splicing patterns in tumors, which in turn influence tumorigenesis and progression [15]. SF3B1, localized on chromosome 2, encodes the largest subunit of the splice factor 3b (SF3b) protein complex. Studies have shown that the SF3B1 complex participates in the formation of U2 small nuclear ribonucleoproteins (snRNPs) by interacting with the 12S subunit and the splice factor 3a (SF3a) complex [16]. SF3B1 mutations are most prevalent in hematologic disorders, particularly myelodysplastic syndromes [17]. Additionally, SF3B1 is highly expressed in ovarian cancer, where its expression negatively correlates with patient prognosis and cytotoxic T-cell infiltration [18]. It also promotes malignancy in pancreatic cancer, endometrial cancer, and glioblastoma [19]. Pla B, a natural macrocyclic lactone analog with antitumor activity, specifically binds to the SF3B complex to inhibit spliceosome function and splicing [20]. Previous studies have demonstrated that Pla B suppresses cell proliferation in multiple cancer types, including cervical cancer, HCC, glioblastoma, chronic lymphocytic leukemia, endometrial cancer, and ovarian cancer [18, 21–24].

Autophagy is a lysosome-dependent self-degradative process and an essential catabolic mechanism in eukaryotic cells [25]. As a form of type II programmed cell death, autophagy and autophagy-related (ATG) proteins play critical roles in cancer progression [26]. It is a multistep process involving the nucleation/initiation, extension, maturation, and recycling of degradative components and membrane compartments [27]. Since the early exploration of autophagy in cancer, accumulating evidence has shown that it exerts dual effects, both promoting and inhibiting cancer growth and progression [28, 29]. The role of autophagy in HCC initiation and development has been well investigated: autophagy can be induced in hepatocytes, and the liver, as a key metabolic organ, underscores the relevance of this process to HCC [30, 31]. The first evidence linking autophagy to hepatocarcinogenesis came from studies on mice expressing a heterozygous Beclin-1 mutant: these mice exhibited impaired autophagy and a high incidence of spontaneous tumors, including liver cancer [32]. Notably, multiple core splicing factors, such as members of the SF3B and SRSF families, as well as SF1 and WTAP, play important roles in HCC [33–37]. While SF3B1 has been targeted with macrolide modulators in various malignancies [38], the specific mechanism by which its inhibitor, Pla B, exerts anti-tumor effects remains a key focus of current research.

Here, we elucidate a critical physiological role of SF3B1 in HCC. First, we observed that SF3B1 expression was significantly upregulated in HCC patients. To dissect SF3B1’s functional relevance in HCC, we employed its specific inhibitor Pla B and investigated its effects through both in vitro and in vivo experiments. Our in vitro findings demonstrated that Pla B treatment effectively inhibited the proliferation of HCC cells while promoting their apoptosis. Integrating RNA sequencing (RNA-Seq) data, we further uncovered that Pla B induces autophagy in HCC cells, and this effect is mediated via the AMPK-mTOR signaling pathway. In our in vivo studies, where nude mice were subjected to subcutaneous HCC tumor implantation followed by intratumoral drug injection, Pla B was shown to suppress tumor growth. Notably, the combined use of Pla B and cisplatin exhibited superior therapeutic efficacy compared to either monotherapy: this combinatorial treatment resulted in reduced tumor proliferation, increased intratumoral apoptosis, and ultimately enhanced cellular autophagy levels.

## Result

### SF3B1 is associated with poor prognosis in HCC

**Fig. 1:**
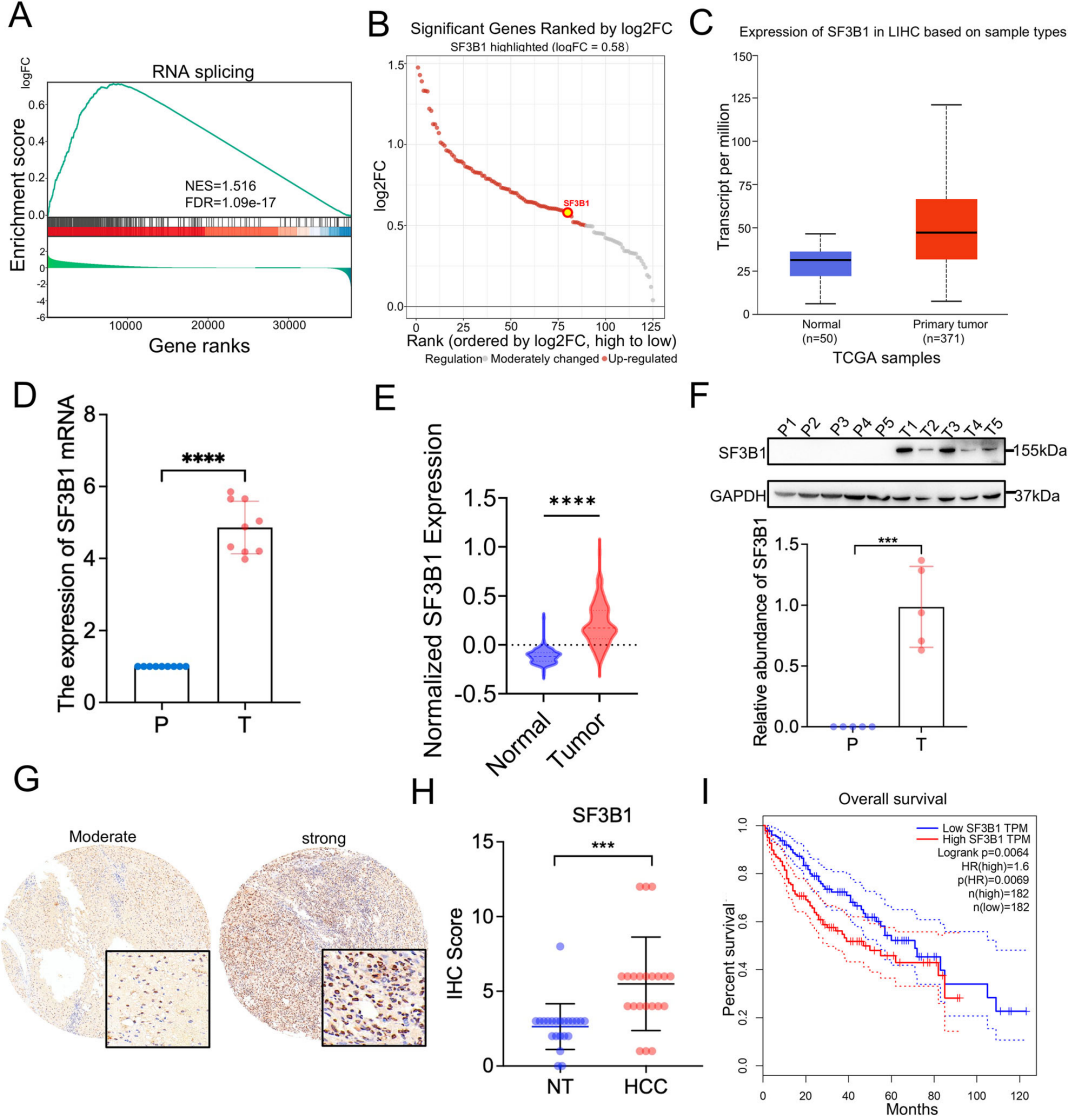
Elevated SF3B1 expression is associated with poor prognosis in HCC. **A** GSEA analysis of splicing-related genes was performed using the gene expression profiles of 50 paired HCC samples from TCGA (NES = 1.516, FDR = 1.09e-17). **B** Gene Ranking Scatter plot of differentially expressed core splicing factors (*n* = 134) between the TCGA-HCC cohort (*n* = 50) and normal tissue in datasets (*n* = 50). **C** SF3B1 mRNA level was analyzed in HCC (*n* = 50) and normal (*n* = 50) samples of the TCGA database. **D** RT-qPCR analysis results of SF3B1 expression in para-tumoral and tumor tissues from 9 HCC patients at the Second Qilu Hospital of Shandong University. **E** SF3B1 protein level was analyzed in HCC (*n* = 50) and normal (*n* = 50) samples of CPTAC database. **F** Western blot results and gray-scale analysis of matched cancerous and non-cancerous tissues from five HCC patients at the Second Qilu Hospital of Shandong University. **G** Representative image of SF3B1 immunohistochemical staining on tissue microarray of HCC patients. **H** Immunohistochemical score of normal samples (*n* = 24) and HCC cancer (*n* = 24). **I** Kaplan-Meier analysis of SF3B1 expression and Overall survival of HCC cancer patients from the Kaplan-Meier Plotter database. All data in the figures (**D**, **E**, **F**, **H**) were presented as mean ± SD. *P*-values were determined by two-tailed unpaired t-tests. *P < 0.05, **P < 0.01, ***P < 0.001.

To identify splicing-related therapeutic targets in HCC, we first integrated transcriptomic data from The Cancer Genome Atlas (TCGA) (50 tumor tissues vs. 50 non-tumor liver tissues) with proteomic profiles from the Clinical Proteomic Tumor Analysis Consortium (CPTAC) (159 tumor tissues vs. 159 matched non-tumor liver tissues). Gene set enrichment analysis (GSEA) of 470 splicing-related genes revealed significant enrichment of RNA splicing pathways in HCC (Fig. 1A, Data S1), with 134 splicing factors (SFs) consistently upregulated at mRNA levels (Fig. 1B, Data S2). Focusing on SF3B1, we analyzed its expression in the TCGA cohort and found it was highly expressed in most HCC samples relative to adjacent non-tumor tissues (Figs. 1C and S1A). To validate this, we collected paired tumor and adjacent non-tumor liver tissues from 9 HCC patients who underwent curative resection at the Second Qilu Hospital. Quantitative real-time PCR (qPCR) confirmed that SF3B1 was frequently overexpressed in HCC tissues (*n* = 9) compared to matched non-tumor tissues (*n* = 9) (Fig. 1D). CPTAC proteomics data further verified a corresponding increase in SF3B1 protein abundance (Fig. 1E, Data S3), and western blot analysis confirmed significantly elevated SF3B1 protein levels in tumor tissues relative to matched non-tumor controls (Figs. 1F and S7). We also constructed a tissue microarray (TMA) containing 24 normal liver tissue samples and 24 HCC specimens; immunohistochemistry (IHC) showed markedly higher SF3B1 protein expression in HCC samples than in normal controls (Fig. 1G, H). Consistently, Kaplan-Meier analysis of the KM Plotter cohort (*n* = 364) revealed a significant association between high SF3B1 mRNA expression and reduced overall survival (OS) (Figs. 1I and S1B). Collectively, multi-omics and histological data demonstrate that SF3B1 is overexpressed in HCC and strongly correlates with aggressive disease biology and poor prognosis, supporting its role as a promising prognostic biomarker and therapeutic target in HCC.

### Inhibition of splicing factor SF3B1 significantly inhibits HCC cells proliferation

**Fig. 2:**
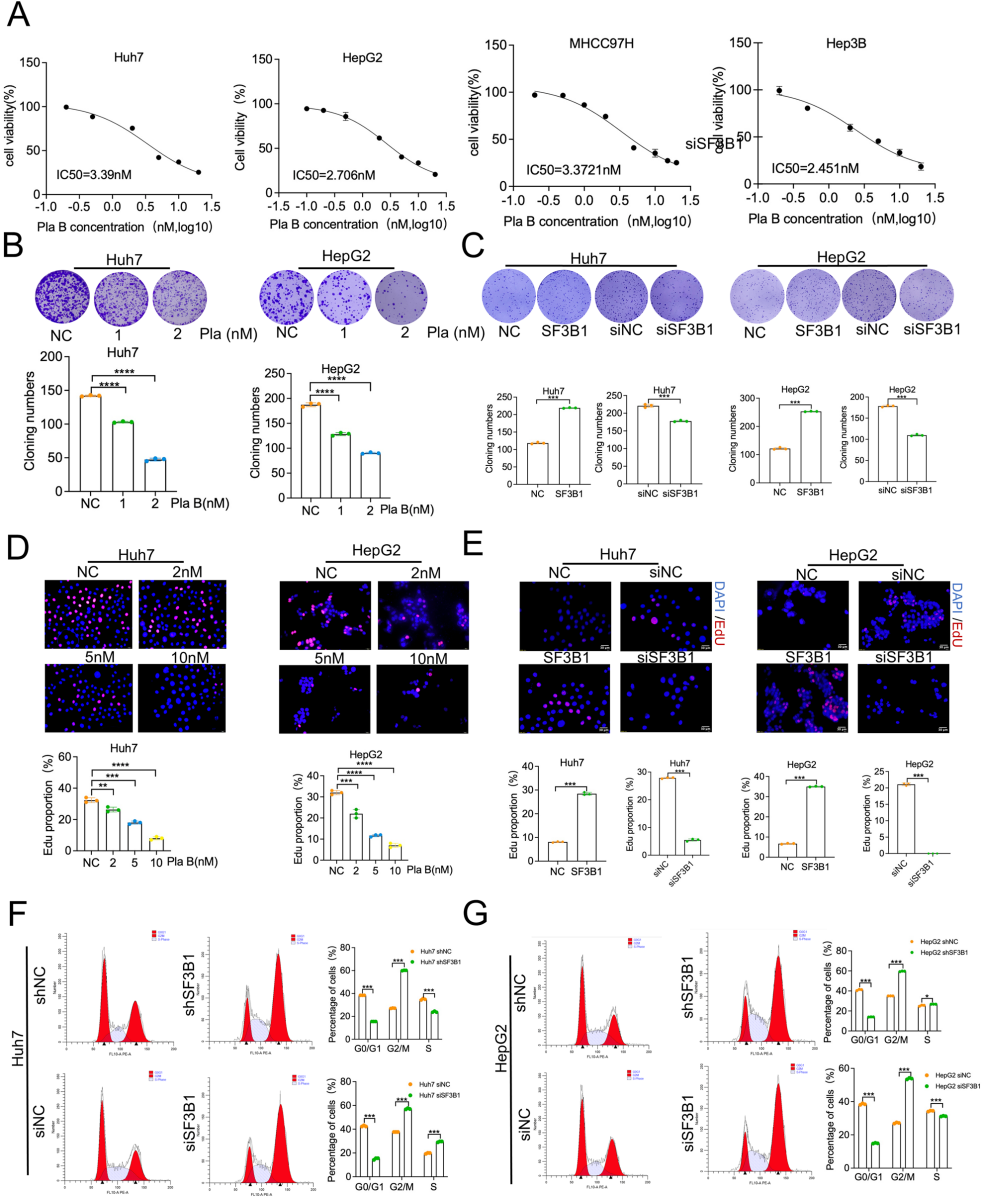
Knockdown of *SF3B1* impairs the proliferation of HCC cells. **A** MTT cell proliferation assay of HCC lines (Huh7, HepG2, Hep3B, and MHCC97H) treated with serial concentrations of Pla B for 48 h, and IC50 determination. **B** Colony formation assay to evaluate Pla B-mediated proliferation inhibition in HCC lines (Huh7and HepG2) following 14-day DMSO/Pla B (1 nM and 2 nM) treatment. **C** Colony formation assay in HCC cells (Huh7 and HepG2) following 14-day treatment with SF3B1 siRNA and SF3B1 overexpression constructs. (*n* = 3 biologically independent experiments). **D** EdU proliferation assay of HCC lines (Huh7 and HepG2) following 48 h treatment with Pla B (2 nM, 5 nM, and 10 nM) and DMSO (control). (EdU assay *n* = 3, all independent biological replicates). **E** EdU proliferation assay in HCC cells (Huh7 and HepG2) following 48 h treatment with SF3B1 siRNA and SF3B1 overexpression constructs. (EdU assay *n* = 3, all independent biological replicates). **F**, **G** Cell cycle distribution analysis by flow cytometry in HCC cells (Huh7 and HepG2) following 48 h treatment with SF3B1 siRNA and SF3B1 shRNA constructs. (*n* = 3 biologically independent experiments). The proportions of G2/M-phase cells were statistically analyzed. All data in the figures **B**–**G** were presented as mean ± SD. *P*-values were determined by two-tailed unpaired t-tests. *P < 0.05, **P < 0.01, ***P < 0.001.

To evaluate the therapeutic potential of Pla B in HCC, we conducted a series of in vitro experiments using four HCC cell lines: Huh7, HepG2, Hep3B, and MHCC97H. MTT assays revealed that Pla B inhibited HCC cell proliferation in a concentration-dependent manner, and IC50 values further verified that high concentrations of Pla B exerted significant growth-suppressive effects (Fig. 2A). Plate colony formation assays provided consistent evidence, showing that even low concentrations of Pla B induced pronounced inhibitory responses in all tested cell lines (Figs. 2B and S2A). Colony formation assays demonstrated that proliferation was significantly attenuated in HCC cells (Huh7 and HepG2) following SF3B1 knockdown (siSF3B1 and shSF3B1), whereas SF3B1 overexpression markedly enhanced the proliferative capacity of HCC cells (Figs. 2C and S2B). The antiproliferative effect of Pla B was further validated by EdU incorporation assays, which showed a progressive reduction in DNA synthesis with increasing concentrations of Pla B (Figs. 2D and S2C). Similarly, EdU assays confirmed that SF3B1 knockdown suppressed proliferation in Huh7 and HepG2 cells, while SF3B1 overexpression promoted cell proliferation (Figs. 2E and S2D).

Furthermore, flow cytometry analysis demonstrated that SF3B1 knockdown induced G2/M phase arrest in Huh7 and HepG2 cells (Fig. 2F, G), thus impeding cell cycle progression and supporting a critical regulatory role of SF3B1 in HCC cell proliferation. Collectively, these results demonstrate that Pla B inhibits the proliferation of HCC cells in vitro in a dose-dependent manner.

### SF3B1 knockdown induces elevated apoptosis in HCC cells

**Fig. 3:**
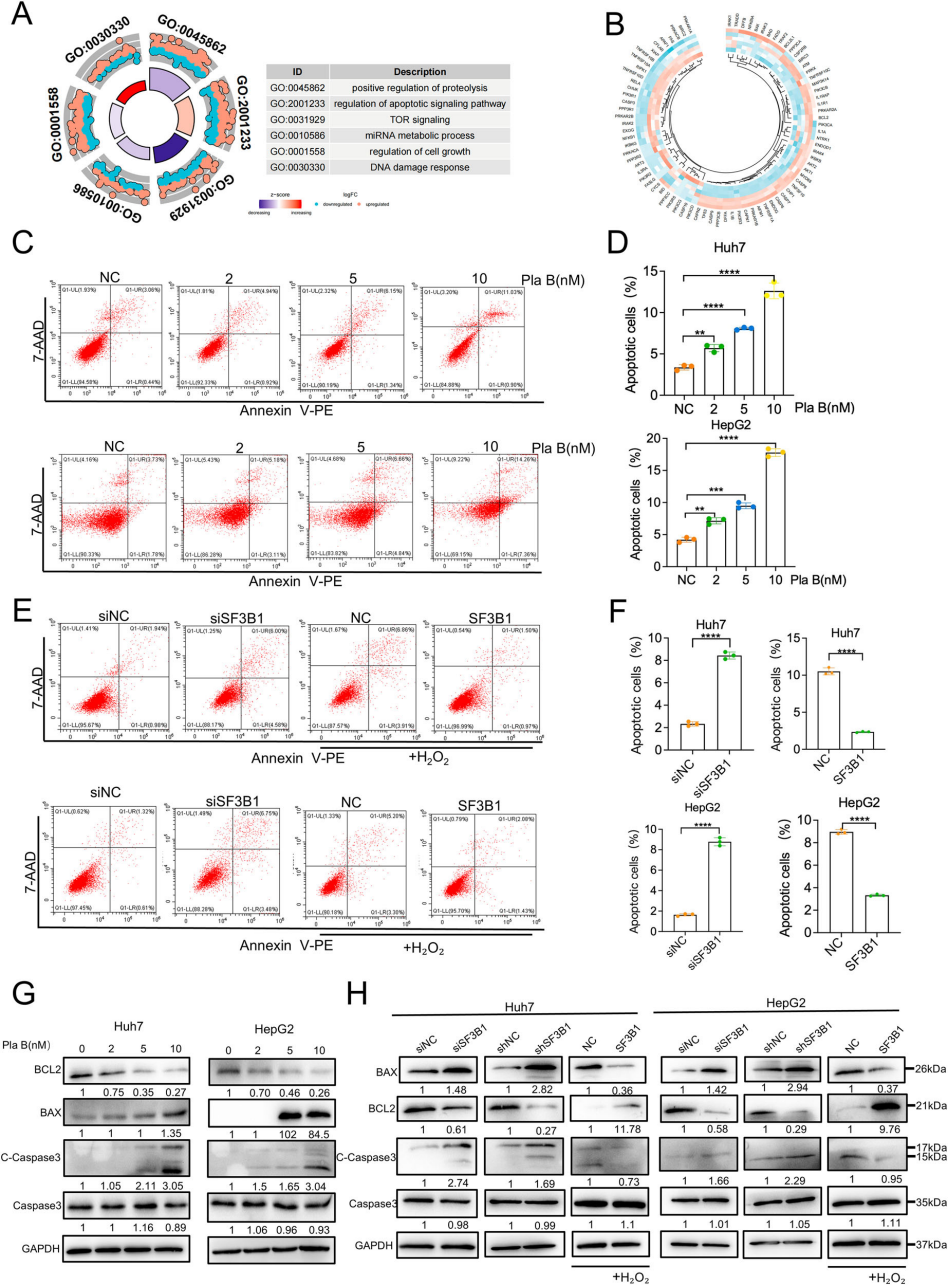
Inhibition of SF3B1 induces apoptosis in HCC cells. **A** GO analysis circular bubble plot from RNA-seq data of Huh7 cells treated with DMSO or Pla B (5 nM) for 48 h (*n* = 3 each group). **B** Circular heatmap of apoptosis-related genes under Pla B (5 nM) treatment for 48 h (*n* = 3 each group). **C**, **D** Apoptotic cells were detected by flow cytometry after staining with Annexin V-PE/7-AAD in HCC cells (Huh7and HepG2) after 48 h treatment with different concentrations of Pla B (*n* = 3 biologically independent experiments). The percentage of apoptotic cells included early and late apoptotic cells. **E**, **F** Apoptotic cell detection via flow cytometry in HCC cells (Huh7 and HepG2) following 48 h treatment with SF3B1 siRNA and SF3B1 overexpression constructs; cells were stained with Annexin V-PE/7-AAD (*n* = 3 biologically independent experiments). The percentage of apoptotic cells included early and late apoptotic cells. **G** Western blot analysis of apoptosis markers after 48 h treatment with different concentrations of Pla B (2 nM, 5 nM, and 10 nM) and DMSO (control) in HCC cells (Huh7and HepG2). **H** Western blot analysis of apoptosis markers after 48 h treatment with SF3B1 siRNA, SF3B1 shRNA, or SF3B1 overexpression constructs in HCC cells (Huh7 and HepG2). All data in the figures (**D**, **F**) were presented as mean ± SD. *P*-values were determined by two-tailed unpaired t-tests. *P < 0.05, **P < 0.01, ***P < 0.001.

Next, we performed GO functional enrichment analysis on differentially expressed genes (DEGs) derived from RNA sequencing of Huh7 cells treated with DMSO or Pla B (5 nM) (Fig. S3A–G). The analysis revealed that apoptosis-related signaling pathways were significantly enriched and ranked among the top terms (Fig. 3A). Moreover, most apoptosis-related genes were upregulated under Pla B treatment, suggesting that the apoptotic process may have been activated (Fig. 3B). Consistent with this hypothesis, flow cytometry analysis showed that Pla B-mediated SF3B1 inhibition significantly induced apoptosis in a dose-dependent manner across multiple HCC cell lines (Huh7, HepG2, Hep3B, and MHCC97H) (Figs. 3C, D, and S3H, I). To further validate the anti-apoptotic function of SF3B1, we transfected HCC cells (Huh7, HepG2, and MHCC97H) with SF3B1 knockdown or overexpression constructs. Flow cytometric analysis subsequently confirmed the aforementioned findings (Figs. 3E, F and S3J–M). To clarify the molecular mechanisms underlying this apoptotic response, we conducted Western blot analysis. Results demonstrated that Pla B treatment significantly upregulated the expression of Cleaved Caspase-3 (a key executor of apoptosis) in all tested HCC cell lines (Figs. 3G and S3N; S7). Moreover, SF3B1 inhibition markedly downregulated the anti-apoptotic protein BCL2 in Huh7 and HepG2 cells, while concurrently upregulating the pro-apoptotic protein BAX in Huh7, HepG2, and Hep3B cells. These results were further validated in HCC cell lines with stable SF3B1 knockdown or overexpression (Figs. 3H, and S3O; S7). Collectively, these findings indicate that SF3B1 inhibition activates the apoptotic pathway in HCC cells, which contributes to its anti-tumor effect.

### Pla B promotes autophagy through the AMPK-mTOR signaling pathway

**Fig. 4:**
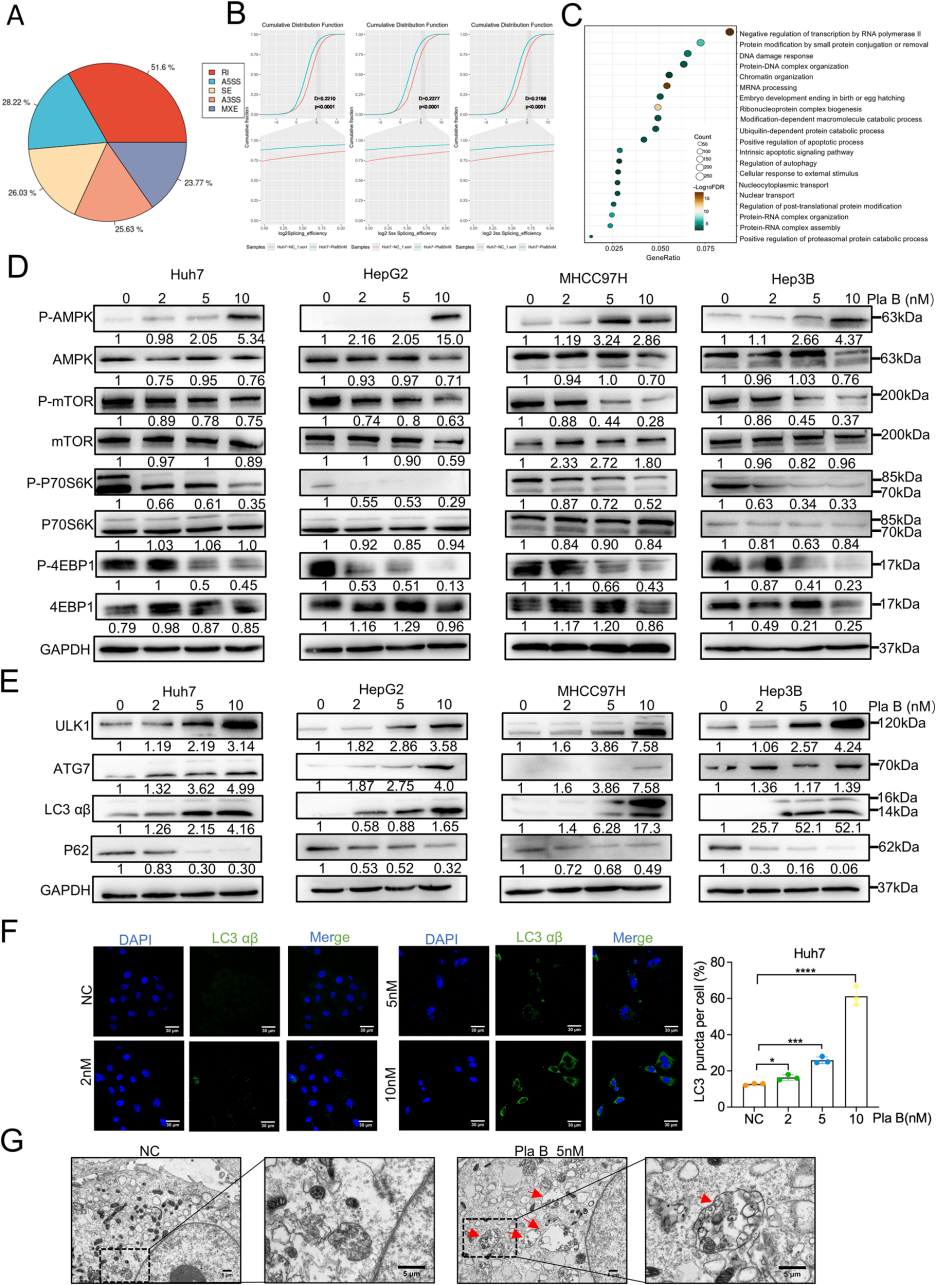
Pla B promotes autophagy through the AMPK-mTOR signaling pathway. **A** Pie chart showing the distribution of AS events identified from RNA-seq data of Huh7 cells treated with DMSO or Pla B (5 nM) for 48 h (*n* = 3 each group). **B** Landscape of splicing efficiency at the 5′ splicing sites and 3′ splicing sites was analyzed with splicing efficiency analysis and annotation of Huh7 cells treated with DMSO or Pla B (5 nM) for 48 h (*n* = 3 each group). **C** Bubble plot of Gene Ontology (GO) biological process enrichment analysis for KEGG in Huh7 cells treated with Pla B for 48 h. **D** Western blot analysis of AMPK-mTOR and **E** autophagy signaling pathway-related proteins in HCC cells (Huh7, HepG2, Hep3B, and MHCC97H) after 48 h treatment with Pla B (2 nM, 5 nM, and 10 nM) and DMSO (control). **F** Immunofluorescence staining of LC3αβ in control and Pla B-treated (5 nM, 48 h) Huh7 cells, with puncta quantification (mean ± SD; *n* = 3). All data were presented as mean ± SD. *P*-values were determined by two-tailed unpaired t-tests. *P < 0.05, **P < 0.01, ***P < 0.001. **G** TEM images showing autophagosome formation in Huh7 cells treated with 5 nM Pla B for 48 h and corresponding control cells. Data are presented as mean ± standard deviation from three independent experiments (*n* = 3).

To investigate alternative splicing (AS) events regulated by SF3B1, we analyzed AS patterns using RNA-Seq data from Huh7 cells treated with 5 nM Pla B or DMSO for 48 h. IR was identified as the predominant AS event following SF3B1 inhibition, accounting for 51.6% of total AS changes (Fig. 4A, Data S4). We further calculated splicing efficiency from the RNA-seq data and found that SF3B1 inhibition significantly reduced the efficiency of total splice sites, as well as 5’ splice sites (5SS) and 3’ splice sites (3SS) individually (Fig. 4B). Additionally, Percent Spliced In (PSI) values for 5SS and 3SS provided further evidence to support this reduction in splicing efficiency (Fig. S4A, Data S5). Notably, IR events constituted 78.3% of the identified splicing aberrations, and the core events in this subset were functionally associated with apoptosis and autophagy regulation (Fig. S4B, C). We assessed the IR status of autophagy- and apoptosis-related genes. We selected the *BIRC5* (Survivin) gene. Survivin, a key member of the inhibitor of apoptosis (IAP) family, plays a central role in HCC tumorigenesis and progression [39]. Survivin is overexpressed in a large number of cancers [40]. Downregulation of *BIRC5* is associated with the induction of apoptosis [41]. Subsequently, performed IGV visualization, semi-quantitative analysis, and qPCR assays on the *BIRC5* gene (Fig. S4D–F). KEGG pathway analysis revealed that the impaired pre-mRNA splicing efficiency caused by SF3B1 inhibition primarily affected two key processes: mRNA processing and the regulation of autophagy (Fig. 4C, Data S6). To validate the involvement of autophagy, we examined the expression of core autophagy-related proteins via western blot. Consistent with the RNA-seq results, Pla B treatment markedly increased the expression of phosphorylated AMPK (P-AMPK) while significantly suppressing phosphorylated mTOR (P-mTOR) levels across multiple HCC cell lines (Huh7, HepG2, Hep3B, and MHCC97H) compared to control groups (Figs. 4D and S8), suggesting that AMPK activation may act as a potential compensatory mechanism. Immunoblotting further confirmed that after 48 h of Pla B treatment, the expression of autophagy-related proteins ULK1 and ATG7, as well as LC3αβ (a specific marker of autophagosome formation), was significantly upregulated; in contrast, the expression of P62 (an autophagy substrate) was markedly decreased (Figs. 4E and S8). The above results were also confirmed in HCC cells (Huh7, HepG2 and MHCC97H) that had undergone SF3B1 knockdown or overexpression constructs treatment (Figs. S4G, S8). Immunofluorescence (IF) analysis of LC3αβ showed that, compared to the negative control (NC) group, the number of autophagic vacuoles (AVs) increased in a dose-dependent manner in Pla B-treated groups (2 nM, 5 nM, and 10 nM). At the 10 nM concentration, the number of AVs was increased by more than 3-fold (Figs. 4F and S4H). Transmission electron microscopy (TEM) analysis further revealed a higher number of autophagosome-like structures in Huh7 cells treated with 5 nM Pla B compared to the NC group (Fig. 4G). Subsequently, we treated cells with three classic autophagy inhibitors (3-MA, bafilomycin A1, and chloroquine), and western blot analysis revealed that these inhibitors effectively abrogated the Pla B-induced elevation in autophagic activity (Fig. S4I, S9). Notably, inhibition of autophagy did not result in a further increase in apoptotic levels (Fig. S4J). Additionally, mTOR activator treatment further demonstrated that Pla B-induced autophagy is specifically mediated through the AMPK-mTOR-ULK1-LC3 axis (Figs. S4K; S9). Collectively, these results demonstrate that inhibiting autophagy mediated by SF3B1 in HCC cells through the AMPK-mTOR signaling pathway.

### Pla B, in combination with cisplatin, inhibits the proliferation and promotes the apoptosis of HCC cells in vivo

**Fig. 5:**
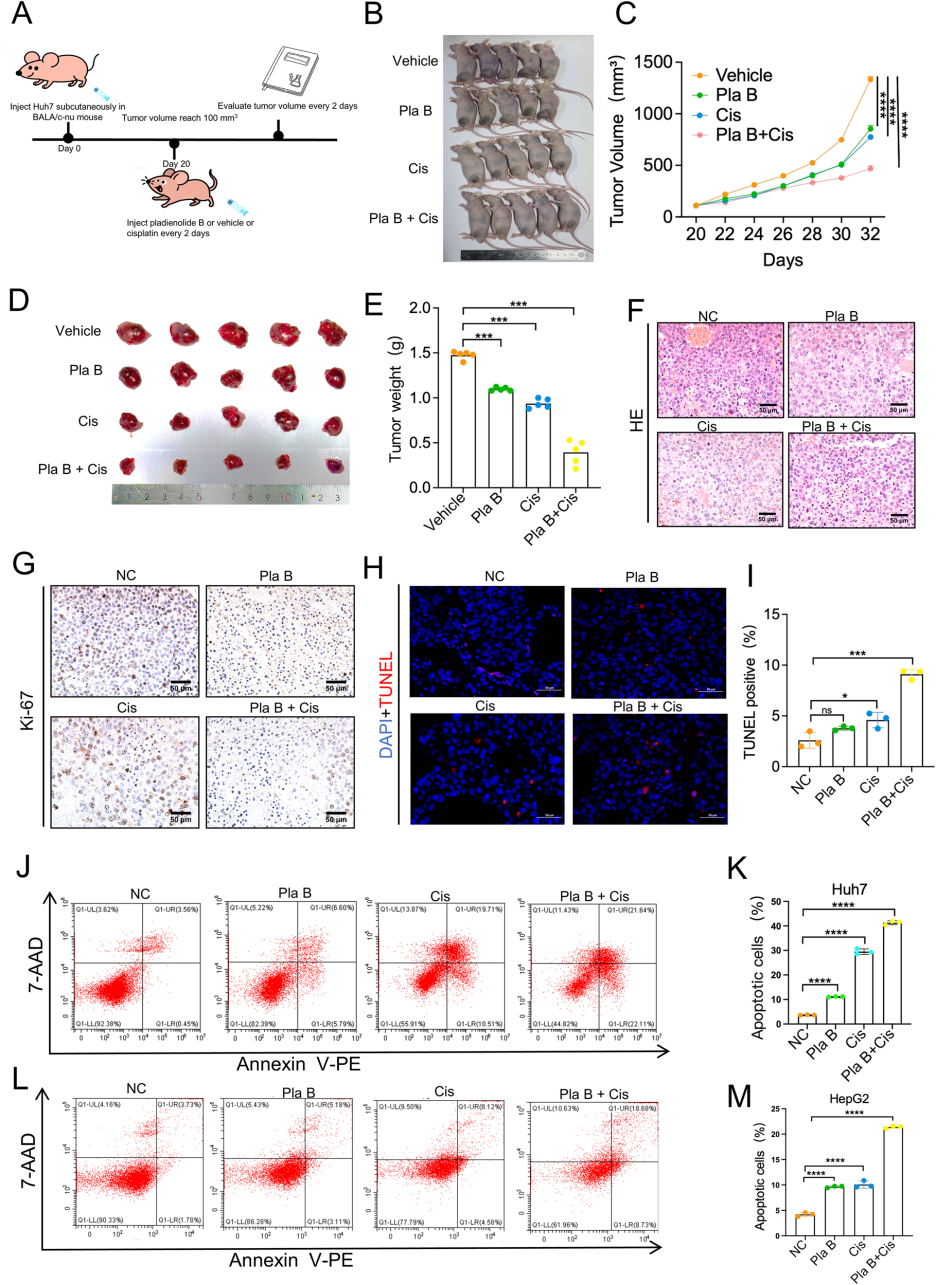
Pla B, in combination with cisplatin, inhibits proliferation and promotes apoptosis of HCC cells in vivo. **A**, **B** Flow chart of mouse tumor inoculation and drug administration. Huh7 cells (2 × 10^5^) were subcutaneously injected into the right leg of BALA/c-nu mice (6–8 weeks old). After 20 days, mice were randomized into treatment cohorts accordingly (*n* = 5 for each group) and given drug treatment: vehicle (10% DMSO in PBS), Pla B (5 mg/kg, every 2days, 10 times), cisplatin (2 mg/kg, every 2days, 10 times)or combined medication. After the tumors reached 100 mm3, tumor volume was evaluated every 2days. Treatment was stopped at day 10, a photograph of a deceased nude mouse. **C** Growth curve of tumor volume. **D** Image of transplantation tumors in vehicle group and Pla B group and cisplatin group and combined medication group. **E** Bar chart of tumor weight of mice in vehicle group and Pla B group. and cisplatin group and Combined medication group. **F** Representative H&E-stained sections of xenograft tumors from control, Pla B-treated, Cis-treated and combination-treated mice. **G** IHC staining for Ki-67 in the indicated groups. Detection (**H**) and quantitative (**I**) analysis of apoptotic cells in tumor sections. **J**–**M** Apoptotic cells were detected by flow cytometry after staining with Annexin V-PE/7-AAD in HCC cells (Huh7 and Hep3B) (after 48 h of combined treatment with Pla B (5 nM), cisplatin(3 μM), or both drugs. (*n* = 3 biologically independent experiments). The percentage of apoptotic cells included early and late apoptotic cells. All data in the figures (**C**, **E**, **I**, **K**, **M**) are presented as mean ± SD. *P*-values were determined by two-tailed unpaired t-tests. *P < 0.05, **P < 0.01, ***P < 0.001.

To evaluate the combined effect of Pla B and cisplatin on tumor growth in vivo, we established a subcutaneous xenograft model by inoculating Huh7 HCC into BALB/c-nu mice. The mice then received intratumoral injections of Pla B, cisplatin, or their combination (Fig. 5A). Results showed that both Pla B and cisplatin suppressed HCC tumor growth, with the combined treatment exerting a more pronounced inhibitory effect. Specifically, SF3B1 inhibition by Pla B, either alone or in combination with cisplatin, significantly reduced tumor volume and weight (Fig. 5B–E). Histological analysis of tissue sections from Huh7-derived xenograft models further demonstrated that Pla B and cisplatin treatments significantly inhibited tumor cell proliferation and enhanced apoptosis, with the combination yielding the strongest effects (Fig. 5F–I). To validate these findings in vitro, we performed complementary experiments. MTT assays confirmed that cisplatin inhibits HCC cell proliferation in a concentration-dependent manner, with IC50 values indicating marked suppression of cell viability at higher concentrations (Fig. S5A). As shown in the combination index (CI), a synergistic effect was observed for the combination of Pla B and Cis (Fig. S5B). Flow cytometry and immunoblotting analyses revealed that the Pla B-cisplatin combination significantly induced apoptosis in multiple HCC cell lines (Huh7, HepG2, MHCC97H and Hep3B), with a more pronounced pro-apoptotic effect than either monotherapy (Figs. 5J–M and S5C–G; S9). In summary, the combination therapy exhibited superior anti-tumor efficacy relative to monotherapies.

### Promotion of autophagy by Pla B in combination with cisplatin

**Fig. 6:**
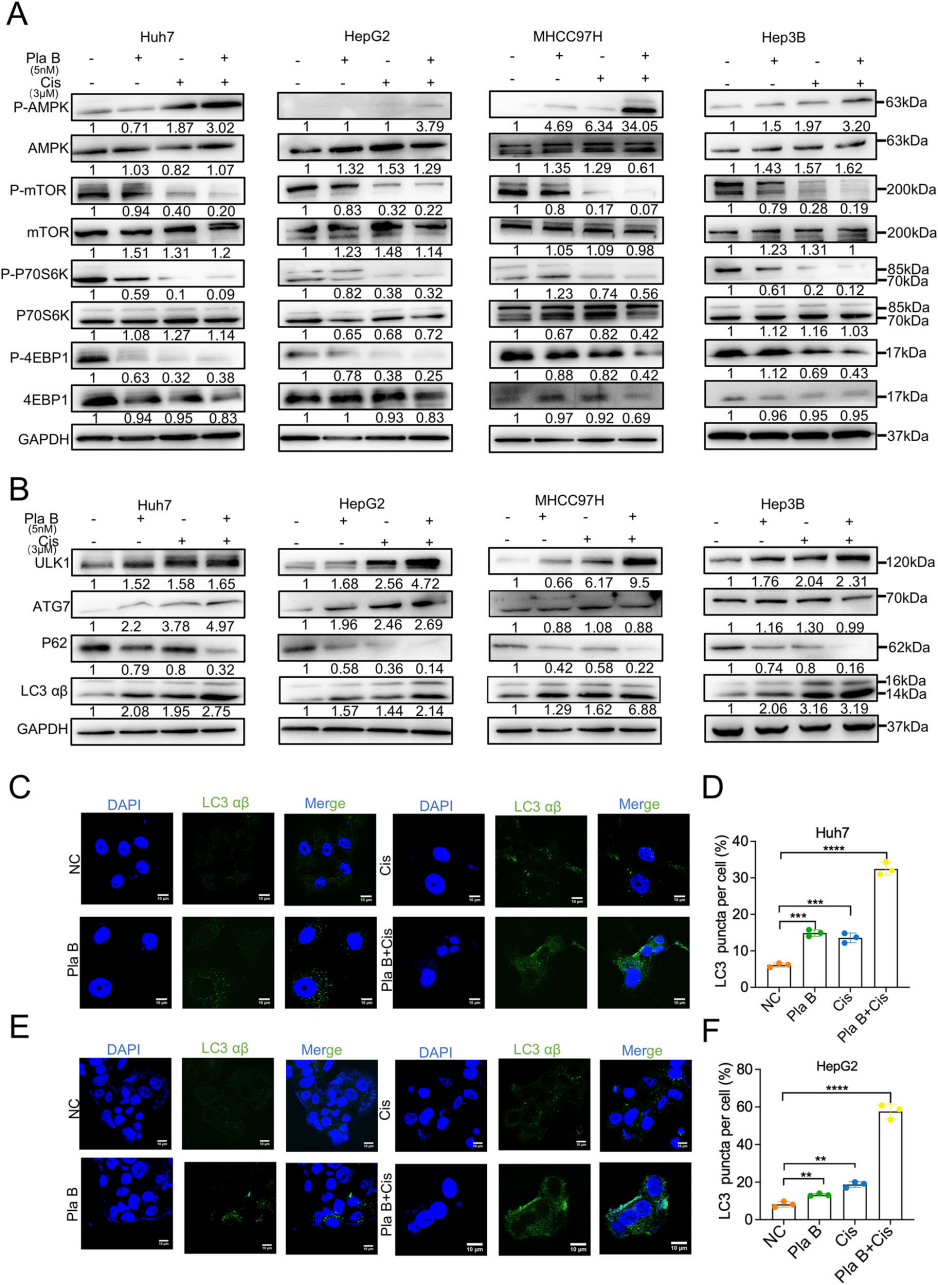
Promotion of autophagy by Pla B in combination with cisplatin. **A** Western blotting detection of AMPK-mTOR signaling pathway-related protein expression levels in HCC cells (Huh7, HepG2, Hep3B, and MHCC97H) after 48 h of Pla B or cisplatin or combination medication cotreatment. **B** Western blotting detection of autophagy signaling pathway-related protein expression levels in HCC cells after 48 h of Pla B (5 nM), or cisplatin(3 μM), or combination medication treatment. **C**–**F** Immunofluorescence staining of LC3αβ in Huh7 and HepG2 cells. Cells were subjected to control treatment, or cotreatment with Pla B (5 nM), or cisplatin(3 μM), or their combination for 48 h, followed by puncta quantification. (mean ± SD; *n* = 3). All data in the figures (**D**, **F**) were presented as mean ± SD. *P*-values were determined by two-tailed unpaired t-tests. *P < 0.05, **P < 0.01, ***P < 0.001.

HCC poses a major global health challenge, continuing to impose a heavy burden on populations worldwide. We hypothesized that the combination of Pla B and cisplatin enhances autophagic activity in HCC. Immunoblotting assays confirmed this hypothesis: the Pla B-cisplatin combination therapy significantly upregulated the expression of phosphorylated AMPK (p-AMPK) while downregulating proteins in the mTOR signaling pathway, including phosphorylated mTOR (P-mTOR) and phosphorylated P70S6K (P-P70S6K) (Figs. 6A and S9). Concurrently, this combinatorial treatment induced the expression of the autophagy-related proteins ULK1 and LC3αβ, and promoted the downregulation of P62 (an autophagy substrate) (Figs. 6B and S9). To further investigate the effects of Pla B and cisplatin, alone or in combination, on autophagy, we performed LC3αβ immunofluorescence assays. The formation of LC3 puncta (a hallmark of autophagosome formation) revealed that the combination treatment elicited a significantly stronger autophagic signal compared to either monotherapy (Fig. 6C–F). This result was further verified by IF staining of LC3αβ in tumor-bearing mouse sections (Fig. S6A, B). Notably, this finding aligns with our earlier observation that the drug combination induces more robust autophagy in tumor cells than individual treatments.

## Discussion

Dysregulated RNA splicing is a molecular hallmark of nearly all tumor types. Cancer-associated splicing abnormalities can drive tumorigenesis through multiple mechanisms [42]. In the present study, we systematically identified and validated the splicing factor SF3B1 as a key oncogenic regulator in hepatocellular carcinoma (HCC), and delineated its mechanistic role in tumorigenesis and progression, as well as its potential as a therapeutic target. First, we screened for survival-related splicing factors in HCC using TCGA data and found that the splicing factor SF3B1 was frequently overexpressed in HCC tissues relative to adjacent non-cancerous tissues. Notably, high SF3B1 expression was associated with poor prognosis in HCC patients; pan-cancer analysis further revealed SF3B1 overexpression across multiple cancer types. These findings were validated using clinical samples: RT-qPCR, immunoblotting, and immunohistochemical analyses consistently confirmed SF3B1 overexpression in HCC, supporting its oncogenic potential and close correlation with unfavorable prognosis in HCC patients.

SF3B1 encodes the largest subunit of the spliceosome factor 3b (SF3B) complex, a core component of the U2 small nuclear ribonucleoprotein (snRNP). It is critical for branch site recognition and essential for the early stages of spliceosome assembly [13]. Hotspot mutations in SF3B1 are linked to poor prognosis in several tumor types and cause global disruption of canonical splicing [43]. Prior studies have reported high-frequency SF3B1 mutations in hematologic malignancies [44, 45], as well as in cutaneous melanoma and breast, pancreatic, lung, and prostate cancers [46–48]. These SF3B1 hotspot mutations are thus considered promising candidate therapeutic targets for cancer treatment [43]. Pla B, an inhibitor of the SF3B1 splicing factor, has previously been shown to suppress cell proliferation in cervical cancer, HCC, glioblastoma, chronic lymphocytic leukemia, endometrial cancer, and ovarian cancer [18, 21, 23, 24, 49]. Consistent with these reports, our study confirms that Pla B inhibits HCC cells' proliferation, as evidenced by MTT assays, plate cloning assays, and EdU labeling experiments. Further, differential gene analysis via RNA-Seq, combined with flow cytometry and western blot experiments, demonstrates that Pla B promotes apoptosis in HCC cells, with the apoptotic rate increasing in a dose-dependent manner as drug concentration rises.

Beyond Pla B, multiple spliceosome inhibitors with distinct mechanisms are under preclinical or clinical development for cancer therapy, targeting core spliceosomal components (e.g., U2 snRNP), modulating AS or interfering with cancer-associated splicing programs. H3B-8800, an orally bioavailable small molecule, binds the SF3b complex directly to compete with Pla B. It potently inhibits spliceosome-mutant hematologic malignancies (e.g., myelodysplastic syndrome, chronic lymphocytic leukemia) and solid tumors (e.g., pancreatic, ovarian cancer), reducing SF3B1-mutant pancreatic cancer cell viability and inducing apoptosis in vitro [50]. Spliceostatin A (SSA) binds the SF3B1 subunit with high affinity, blocking U2 snRNP-pre-mRNA interaction and early spliceosomal assembly, thus causing global splicing dysregulation and cervical cancer cell cycle arrest [51]. It exerts significant antitumor effects in breast cancer models (especially in aberrant splicing-dependent cancer cells) [52]. Its derivative E7107 once entered Phase I clinical trials for solid tumors but was suspended due to dose-limiting toxicities (e.g., myelosuppression), with structural optimization ongoing to improve safety.

Analysis of AS events in RNA-Seq data revealed that Pla B treatment significantly affects IR in a large number of genes, a change that may contribute to transcriptional instability. Notably, splicing efficiency at both 3’ splice sites (3’SS) and 5’ splice sites (5’SS), as well as overall splicing efficiency, was markedly reduced, indicating widespread aberrant pre-mRNA splicing following Pla B exposure. Furthermore, KEGG pathway enrichment analysis of DEGs identified significant enrichment in autophagy-related signaling pathways. Autophagy is a key cellular homeostatic process that facilitates the degradation and recycling of cellular components [26]. As a form of type II programmed cell death, autophagy and autophagy-related (ATG) proteins play critical roles in cancer progression [53]; however, dysregulated autophagy can drive the pathogenesis of diseases, including cancer [54]. In our study, we observed that with increasing Pla B concentration, the level of LC3αβ (a marker of autophagy flux initiation) was elevated, while the expression of P62 (an indicator of autophagic degradation) was reduced. These changes collectively demonstrate that Pla B can induce autophagy in HCC cells.

Based on the above findings, we investigated the upstream signaling pathways driving autophagy induction. The AMPK/mTOR/ULK1 pathway acts as a key regulator of autophagy, mediating the balance between autophagy initiation and inhibition during cellular stress responses [55]. Energy deprivation activates AMP-activated protein kinase (AMPK), a cellular energy sensor, thereby triggering autophagy [56]. Under energy stress, AMPK activation regulates autophagy through the phosphorylation of ULK1 [57]. In contrast, autophagy is inhibited by mammalian target of mTOR, a central regulator of cellular growth that integrates signals from growth factors and nutrients [58]. Our study demonstrates that Pla B treatment activates the expression of phosphorylated AMPK (P-AMPK) in HCC cells. This activation of P-AMPK further inhibits the expression of phosphorylated proteins related to the mTOR pathway, including P-mTOR, P-P70S6K, and P-4EBP1, which in turn triggers the activation of ULK1. Ultimately, this signaling cascade modulates the expression of key autophagy-related proteins: ATG7, LC3αβ, and P62. Two complementary approaches confirmed that Pla B induces autophagy in HCC cells: immunofluorescence staining of LC3αβ (a marker that reflects autophagosome formation) and transmission electron microscopic observation of autophagosome-like structures. Notably, this regulatory pattern is conserved in other spliceosome inhibitors: Isoginkgetin, a natural flavonoid spliceosome inhibitor that blocks U4/U6. U5 tri-snRNP integration and subsequent spliceosome activation potently induce AMPK-ULK1-mediated cytotoxic autophagy in HCC cells [59]. Specifically, activated AMPK directly phosphorylates ULK1 and concurrently inhibits the mTORC1 signaling pathway, thereby relieving mTORC1-mediated negative regulation of the ULK1 complex, initiating autophagosome formation and ultimately promoting tumor cell death.

Acquired resistance to cisplatin is a well-documented challenge in cancer therapy, and the long-term efficacy of its widespread clinical application remains controversial. To address this, we validated the synergistic effect of Pla B and cisplatin using a murine HCC xenograft model. In vivo experiments demonstrated that the Pla B-cisplatin combination enhanced inhibition of HCC growth, suppressed tumor cell proliferation, and promoted tumor cell apoptosis, findings that were further corroborated by complementary in vitro cell-based assays. Notably, we made a striking observation: the drug combination induced significantly stronger autophagy in tumor cells compared to either monotherapy. Collectively, these results indicate that pharmacological targeting of SF3B1 with Pla B may be a promising strategy to enhance cisplatin efficacy. Our study provides preclinical evidence supporting the potential of SF3B1 inhibition combined with cisplatin for HCC therapy. However, it is important to note that our findings are currently limited to murine liver cancer models; further validation using patient-derived xenograft (PDX) models, which better recapitulate human tumor biology, remains to be conducted.

In conclusion, our study identifies SF3B1 as an oncogenic splicing factor and a potential prognostic marker in HCC. Mechanistically, we show that an SF3B1 inhibitor induces autophagy in HCC cells by regulating splicing events that converge on the AMPK/mTOR/ULK1 pathway. Furthermore, we highlight the significant potential of combining an SF3B1 inhibitor with cisplatin as a therapeutic strategy for HCC.

These findings highlight the potential therapeutic value of combined treatment with Pla B and cisplatin for HCC. This combination lies in its potential for dose reduction and synergistic enhancement. Our in vitro/in vivo data suggest that Pla B sensitizes cancer cells to cisplatin, potentially allowing for the use of lower doses of cisplatin to achieve the same therapeutic effect. This “chemo-sensitizing” strategy is a clinically viable approach to mitigate the dose-limiting toxicities (such as nephrotoxicity and neurotoxicity) associated with high-dose cisplatin therapy. Future translational steps should include rigorous in vivo toxicology studies in higher animal models, with a focus on renal and hepatic function, as well as the evaluation of optimal dosing schedules to maximize the therapeutic window.

This study has several limitations that merit consideration. First, the number of clinical samples used for experimental validation is relatively small. Although our findings are supported by TCGA dataset analysis and GSEA enrichment studies that demonstrated significant SF3B1 enrichment in HCC, as well as HCC tissue microarray analysis that confirmed high SF3B1 protein expression, the clinical sample-based validation in this study only served as supplementary evidence for these bioinformatic and tissue microarray data. This limitation will be addressed in future studies by expanding the clinical sample cohort to further strengthen the clinical relevance of our findings.

In conclusion, our study identifies SF3B1 as an oncogenic splicing factor and a potential prognostic marker in HCC. Mechanistically, we demonstrate that SF3B1 inhibition by Pla B modulates splicing events that converge on the AMPK/mTOR/ULK1 signaling pathway, thereby inducing autophagy in HCC cells. Furthermore, we provide preclinical evidence for the significant potential of combining the SF3B1 inhibitor Pla B with cisplatin as a novel therapeutic strategy for HCC. These findings not only deepen our understanding of SF3B1-mediated oncogenesis in HCC but also lay a foundation for the development of spliceosome-targeted therapies for HCC treatment.

## Materials and methods

### Cell lines, cell culture and drugs

HCC cell lines Huh7, HepG2, Hep3B, and MHCC97H were kindly provided by Prof. Jian-Zhao Liu (Shandong University, Jinan, China). The identity of all cell lines was verified by short tandem repeat (STR) profiling. Huh7, HepG2, and MHCC97H cells were maintained in Dulbecco’s modified Eagle’s medium (DMEM; Gibco, Thermo Fisher Scientific) supplemented with 10% (v/v) fetal bovine serum (FBS; Gibco) and 1% penicillin–streptomycin (Macgene). Hep3B cells were cultured in Minimum Essential Medium (MEM; Macgene) containing 10% FBS, 1% penicillin-streptomycin, and 1% non-essential amino acids. All cell cultures were incubated at 37 °C in a humidified atmosphere with 5% CO₂. Pla B (sc-391691) was purchased from Santa Cruz and dissolved in DMSO. Cisplatin was purchased from TargetMol and dissolved in PBS. Cells were treated with DMSO, Pla B(5 nM,), 3-MA (5 mM), and Pla B(5 nM,) + 3-MA (5 mM), 48 h post-treatment, Western blot analysis was performed to examine the expression alterations of autophagy-related marker proteins. According to the above procedures, conduct the tests for bafilomycin A1 (5 nM) and chloroquine (10 μM, 48 h).

### Human liver samples

Para-tumoral and tumor tissue samples of liver cancer were collected from patients who underwent treatment at the Department of Hepatobiliary Surgery, the Second Qilu Hospital of Shandong University. Fresh tissue specimens were obtained within 2 h after surgery, cut into 5-mm pieces, and placed in cryopreservation tubes. All tissue samples were stored at –80 °C. All patients provided written informed consent, and the study was approved by the Ethics Committee of Shandong University (Approval No.: KYLL-2023-279).

### Nude mouse xenograft model

The Animal Ethics Research Committee of Shandong University specifies a maximum allowable tumor diameter of 15 mm. All animal experiments were approved by the Animal Experiment Ethics Committee (KYLL-2023-014) of the Second Qilu Hospital of Shandong University, and no tumors exceeded this limit throughout the study. Male BALB/c-nude mice (6–8 weeks old) were purchased from Beijing VTLU Co., Ltd. The required sample size of mice was estimated using the resource equation method; a total of 32 mice were initially used to establish subcutaneous tumor models by inoculation with 2 × 10⁶ Huh7 cells. Twenty days after injection, 20 mice with tumor volumes of approximately 100 mm³ and minimal variation were selected, and 12 mice with excessively large or small tumors were excluded. The 20 eligible mice were randomly divided into 4 groups (with 5 mice in each group) by the researchers: vehicle (10% DMSO, once daily), Pla B (0.5 mg/kg, every 3 days for six times) [21, 60], cisplatin (2 mg/kg, every 3 days for six times), and combination therapy. A single-blind design was adopted for all animal experiments. Another group of researchers responsible for tumor volume measurement and experimental result evaluation was unaware of the mice’s grouping and treatment protocols. At the end of the experiment, mice were anesthetized to measure tumor volume and weight. The tumor volume was calculated using the formula: V = (length × width²)/2. After euthanasia, the mice were immediately dissected to collect tumor tissue samples. Samples were either rapidly frozen or fixed prior to sectioning for histopathological analysis. A professional pathologist then performed hematoxylin-eosin (HE) staining, TUNEL apoptosis assay, and Ki-67 nuclear staining on the sections to evaluate tumor cell proliferation and apoptosis.

### Colony formation assay

Huh7, HepG2, Hep3B, and MHCC97H cells were seeded into six-well plates at a density of 400 cells per well, followed by treatment with DMSO and Pla B (1 nM and 2 nM) at the indicated concentrations. After 2 weeks of incubation, cells were fixed with methanol and stained with 0.1% crystal violet. Cell colonies were counted for quantification. Colony formation assays were performed with *n* = 3 biological replicates (each with 3 technical replicates) using independently seeded cell cultures. Data are presented as the mean ± standard deviation (SD) and are representative of three independent experiments.

### Western blot

Protein extraction and western blot analysis were performed as follows: Samples were lysed on ice using Western and IP Cell Lysis Buffer, and protein concentrations were determined via the bicinchoninic acid (BCA) protein assay kit (Beyotime). Protein samples were separated by sodium dodecyl sulfate-polyacrylamide gel electrophoresis (SDS-PAGE) and transferred to membranes. The membranes were blocked with 5% skim milk, then incubated overnight at 4 °C with primary antibodies (all diluted at 1:1000). After washing, the membranes were incubated with horseradish peroxidase (HRP)-conjugated secondary antibodies (1:10,000 dilution, Jackson ImmunoResearch). Specific proteins were detected using an electrochemiluminescence (ECL) system (GE Healthcare, UK). Antibodies in this study included: LC3A/B (D3U4C) XP (Cell Signaling Technology 12741), SQSTM1/62 (Cell Signaling Technology 5114), ATG7 (Cell Signaling Technology 8558), ULK1 (D8H5) (Cell Signaling Technology 8054), Caspase-3 (Cell Signaling Technology 9662), Cleaved Caspase-3 (Cell Signaling Technology 9661), anti-P70S6K (Cell Signaling Technology, 2708), anti-phospho-p70S6K (Cell Signaling Technology, 9234), anti-mTOR (Signaling Technology, 2972), anti-phospho-mTOR (Cell Signaling Technology, 5536), anti-4EBP1 (Cell Signaling Technology, 9452),anti-P-4EBP1 (Thr37/46, Cell Signaling Technology, 9459 S),anti-P-AMPK (40H9) (Thr172, Cell Signaling Technology, 2535 S),anti-AMPK (Cell Signaling Technology, 2535). Western blot analyses were repeated for *n* = 3 biological replicates (independent cell lysates or tissue homogenates), with each blot quantified 3 times as technical replicates. Representative blots are shown, and gray-scale analysis was conducted.

### Transmission electron microscopy

Huh-7 cells were treated with Pla B for 48 h, then harvested and fixed for transmission electron microscopy (TEM) analysis. Sample fixation, embedding, sectioning, and staining were performed by Servicebio Biotechnology Co., Ltd. (Wuhan, China). Ultrastructural observation was conducted using a transmission electron microscope, and images were captured to evaluate autophagosome formation.

### Flow cytometry

Apoptosis was analyzed by flow cytometry using the Annexin V-PE/7-AAD Apoptosis Detection Kit (Vazyme, A213-01). For the Pla B-induced apoptosis assay, cells were treated with Pla B at final concentrations of 0, 2, 5, and 10 nM for 48 h. Cells were digested with EDTA-free trypsin (Macgene, CC035) for 3 min, collected by centrifugation, washed with cold phosphate-buffered saline (PBS), and resuspended in 100 μL of 1× binding buffer at a density of 5 × 10⁵ cells/mL. Next, 5 μL of Annexin V-PE and 5 μL of 7-AAD were added, and the mixture was incubated for 10 min in the dark. After adding an additional 400 μL of 1× binding buffer, the cell suspension was filtered through a 0.22 μm membrane and analyzed using a CytoFLEX S flow cytometer (Beckman Coulter Life Science) within 20 min. A minimum of 1 × 10⁴ cells were collected per sample to quantify the apoptotic rate. Data were analyzed with CytExpert software. Early apoptotic cells were defined as Annexin V-PE single-positive (Annexin V-PE⁺/7-AAD⁻), while late apoptotic cells were Annexin V-PE/7-AAD double-positive (Annexin V-PE⁺/7-AAD⁺). Apoptosis rate calculation method: Total apoptosis rate = percentage of early apoptotic cells (Q1-LR) + percentage of late apoptotic cells (Q1-UR), and the total apoptosis rate was used for statistical comparison between groups.

### Cell proliferation assays

Cell viability and proliferation were assessed using the methyl-thiazolyl diphenyl-tetrazolium bromide (MTT) assay. Cells were seeded in 96-well plates at a density of 1–4 × 10³ cells per well, allowing the cells to adhere to the culture surface and enter the exponential growth phase. After culturing for the specified time, 10 μL of MTT solution (5 mg/mL) was added to each well, followed by incubation at 37 °C for 4 h. Cell growth was monitored over 5 consecutive days, and the half-maximal inhibitory concentration (IC₅₀) was calculated 48 h post-treatment. After centrifugation, the supernatant was discarded, and 100 μL of dimethyl sulfoxide (DMSO) was added to each well to dissolve the formazan crystals. Absorbance was measured at 570 nm using a microplate reader (Bio-Rad, Hercules, CA, USA). Cell proliferation was further evaluated via the EdU incorporation assay using the Cell-Light EdU Apollo567 In Vitro Kit, performed according to the manufacturer’s instructions. Briefly, cells were seeded on glass coverslips in 24-well plates at a density of 2–4 × 10⁴ cells per well. After attachment, cells were incubated with culture medium containing EdU for 20–30 min. Subsequently, cells were fixed, stained with Apollo567 fluorescent dye (to detect EdU-positive proliferating cells), and counterstained with Hoechst 33342 (to label cell nuclei). MTT assays were performed with *n* = 3 biological replicates (each with three technical replicates) using independently seeded cell cultures.

### Immunofluorescence

After 48 h of Pla B treatment, Huh7 cells were fixed, permeabilized, and blocked sequentially. The cells were then incubated overnight at 4 °C with the primary antibody against LC3A/B (D3U4C) XP (Cell Signaling Technology, 12741). Following thorough washing, a fluorescent dye-conjugated secondary antibody was applied for detection. Cell nuclei were counterstained with DAPI, and images were captured using a confocal laser scanning microscope. LC3 puncta quantification was performed using ImageJ software (version 1.54 f; National Institutes of Health, Bethesda, MD, USA). For each experimental condition, multiple non-overlapping visual fields were randomly selected from three independent biological replicates. A minimum of 1000 morphologically intact hepatocellular carcinoma cells were analyzed per experimental group to ensure statistical power. For groups with reduced cell density induced by drug treatment, the number of imaged fields was appropriately increased to satisfy the predetermined total cell count requirement. Immunofluorescence assays were performed with *n* = 3 biological replicates (each with three technical replicates) using independently seeded cell cultures.

### RT-qPCR

Total RNA was extracted using TRIzol reagent following the manufacturer’s protocol. Complementary DNA (cDNA) was synthesized via reverse transcription using the HiScript II Q RT SuperMix for qPCR Kit (Vazyme, cat# R233-01). Quantitative real-time PCR (qPCR) was performed with SYBR qPCR Pre-mix (Vazyme, cat# Q711-02) on a real-time PCR system. Relative gene expression levels were calculated using the 2^(-ΔΔCt) method and normalized to glyceraldehyde-3-phosphate dehydrogenase *(GAPDH*) as an internal reference gene. *GAPDH* primer sequence r: GGCTGTTGTCATACTTCTCATGG; primer sequence f: GACTTTAAGGGTTACCTGGGTTG. *SF3B1* primer sequence r: GCCCACTCCTTGAGCTTCAT; primer sequence f: CGTCTGTGTGTTCGAGTGGA. *BIRC5* primer sequence r: GTCTGGCTCGTTCTCAGTGG; f: CTTTCTCAAGGACCACCGCA. RT-qPCR assays were performed with *n* = 3 biological replicates (each with 3 technical replicates) using independently seeded cell cultures.

### RNA-Seq analysis

Raw data have been deposited in the National Genomics Data Center (https://ngdc.cncb.ac.cn/bioproject/) under the accession number PRJCA054265. Huh7 cells treated with DMSO or Pla B (5nM) for 48h were collected; each group of samples contained triplicates, and each condition was an independent biological replicate. Next-generation sequencing was performed by Ribobio Biotechnology Company. Raw paired-end FASTQ files were quality-checked using FastQC, then trimmed with Trim Galore (v0.6.10) using default Illumina adapters (quality threshold Q ≥ 20, minimum read length 20). The essential QC metrics are shown in Fig. S2A–G. Post-trimming quality control was re-evaluated with FastQC. Reads were aligned to the human reference genome (GENCODE release v42) using HISAT2 (v2.2.1) with default paired-end parameters. SAM files were converted to BAM format, coordinate-sorted, and indexed using Samtools (v1.9.0). For gene quantification: Gene-level counts were generated using featureCounts (Subread v2.0.2) with the same GTF annotation. Raw counts were analyzed in R (v4.4.1) using DESeq2 (v1.48.2). We incorporated the batch variable as a covariate in the DESeq2 design formula (e.g., design = ~ batch + condition), which allows DESeq2 to model and account for batch-related variation during differential expression analysis. This approach effectively controls for batch effects while estimating the effect of the experimental condition of interest. Low-count genes were filtered out (retaining those with row sums ≥ 10). Size factors and dispersion were estimated using default settings, and Wald tests were applied for group comparisons. P-values were adjusted using the Benjamini–Hochberg method; genes with a false discovery rate (FDR) < 0.05 and absolute log2 fold change (|log2FC | ) ≥ 1 were considered differentially expressed. Differentially expressed gene sets were subjected to over-representation analysis and gene set enrichment analysis (GSEA) using clusterProfiler (v4.16.0) against Gene Ontology (GO) Biological Processes and KEGG pathways (with org. eg. db). BioEnricher (v0.1.0) was additionally used for complementary pathway and GO enrichment analyses. Data visualization was performed using ggplot2 (v3.5.2). Alternative splicing events were analyzed using rMATS (v4.3.0, turbo mode) with BAM files and the same GTF annotation, specifying read length and library type. Significant events (skipped exons [SE], alternative 5’ splice sites [A5SS], alternative 3’ splice sites [A3SS], mutually exclusive exons [MXE], and intron retention [IR]) were defined as those with FDR < 0.05 and absolute change in percent spliced in (|ΔPSI| ) ≥ 0.1. Splicing efficiency and IR were quantified using SEAA (v0.9.4) with aligned BAM files and the annotation GTF. We uniformly used FPKM (Fragments Per Kilobase Million) for analysis. The calculation formula is as follows: FPKM = C/(N×L), C = number of fragments mapped to a gene, N = total number of mapped fragments in the sample, L = length of the gene in base pairs (bp).

### Statistics and reproducibility

All data are presented as the mean ± standard deviation (SD) and were subjected to statistical analysis using Student’s two-tailed *t* test or two-way analysis of variance (ANOVA). All experimental results were replicated at least three times with independent samples. A *p*-value < 0.05 was considered statistically significant. Asterisks in the figures indicate statistical significance, as follows: *p < 0.05, **p < 0.01, ***p < 0.001. “ns” denotes non-significance.

## Supplementary information

Supplementary Figures and legends

## Data Availability

All data associated with this study are available in the main text or the supplementary materials. Further information and requests for resources and reagents should be directed to and will be fulfilled by the lead contact, Yuliang Li (lyl.pro@sdu.edu.cn).
