# Supplementary material for: Synergistic effect of Pladienolide B and cisplatin: enhancing autophagy in hepatoma cells through the AMPK/mTOR/ULK1 pathway
Source: Cell Death Discov. 2026 May 8;12:288. doi: 10.1038/s41420-026-03144-5 (PMC13319244; doi:10.1038/s41420-026-03144-5)
Supplement: Supplementary file 1 — Supplementary Figures and legends [file 41420_2026_3144_MOESM1_ESM.docx]

**Synergistic Effect of Pladienolide B and Cisplatin: Enhancing Autophagy in Hepatoma Cells Through the AMPK/mTOR/ULK1 Pathway**

Wei Xiao^1^, Lei Yang^4^, Ze Li^1^, Wujie Wang^2,3^, Zhaojian Liu^2,3,4^, Bin Liu^2,3^ **^🖂^**, Junchao Qin^2,3,4^**^🖂^**,Yuliang Li^1,2,3^**^🖂^**

^1^ School of Clinical Medicine, Hebei University of Engineering, Handan, Hebei, China; ^2^ Department of Interventional Medicine and Minimally Invasive Oncology, The Second Qilu Hospital of Shandong University, Jinan, Shandong, China; ^3^ Institute of Interventional Oncology, Shandong University, Jinan, Shandong, China; ^4^ Ministry of Education, Department of Cell Biology, School of Basic Medical Sciences, Shandong University, Jinan, Shandong, China;


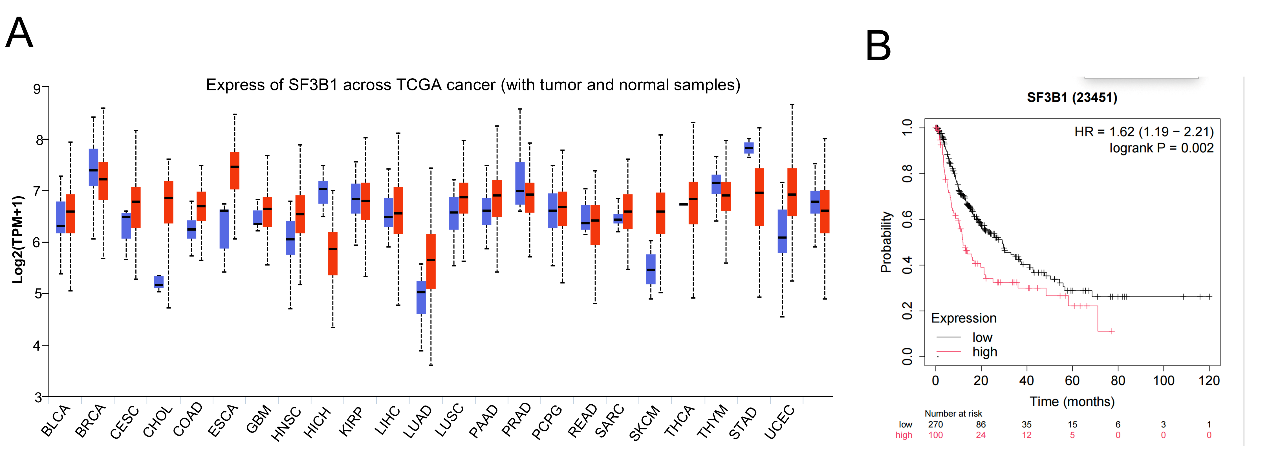


**Figure S1.** **The expression of SF3B1 in TCGA database.** (A) Expression of SF3B1 in the UALCAN (Including Tumor and Normal Samples of TCGA). (B) Patients were divided into low-expression (n = 270) and high-expression (n = 100) groups. High SF3B1 expression was significantly associated with poorer overall survival (HR = 1.62, 95% CI: 1.19–2.21, log-rank P = 0.002).


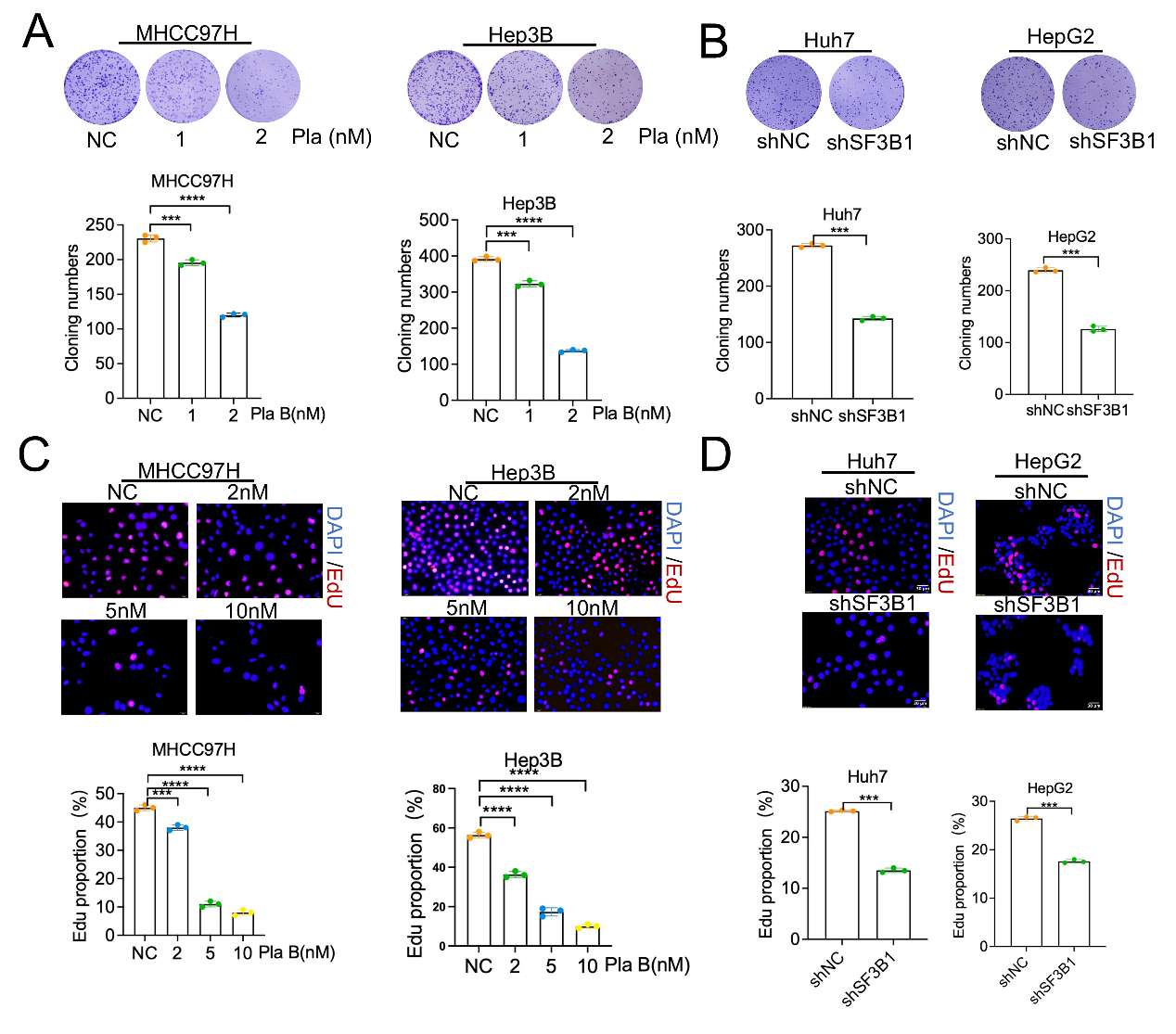


**Figure S2.** **Pla B impairs the proliferation of MHCC97H and Hep3B cells.** (A) Colony formation assay to evaluate Pla B-mediated proliferation inhibition in HCC lines (MHCC97H and Hep3B) following 14-day DMSO/Pla B (1nM and 2 nM) treatment. (B) Colony formation assay in HCC cells (Huh7 and HepG2) following 14-day treatment with shSF3B1 constructs. (n = 3 biologically independent experiments). (C) EdU proliferation assay of HCC lines (MHCC97H and Hep3B) following 48 h treatment with Pla B (2nM, 5nM, and 10 nM) and DMSO (control). (EdU assay n = 3, all independent biological replicates). (D) EdU proliferation assay in HCC cells (Huh7 and HepG2) following 48 h treatment with shSF3B1 constructs. (EdU assay n = 3, all independent biological replicates). All data in the figures were presented as mean ± SD. P-values were obtained via two-tailed unpaired t-tests, with results expressed as mean ± standard deviation (SD). Statistical significance is denoted as follows: **p* < 0.05, ***p* < 0.01, ****p* < 0.001.


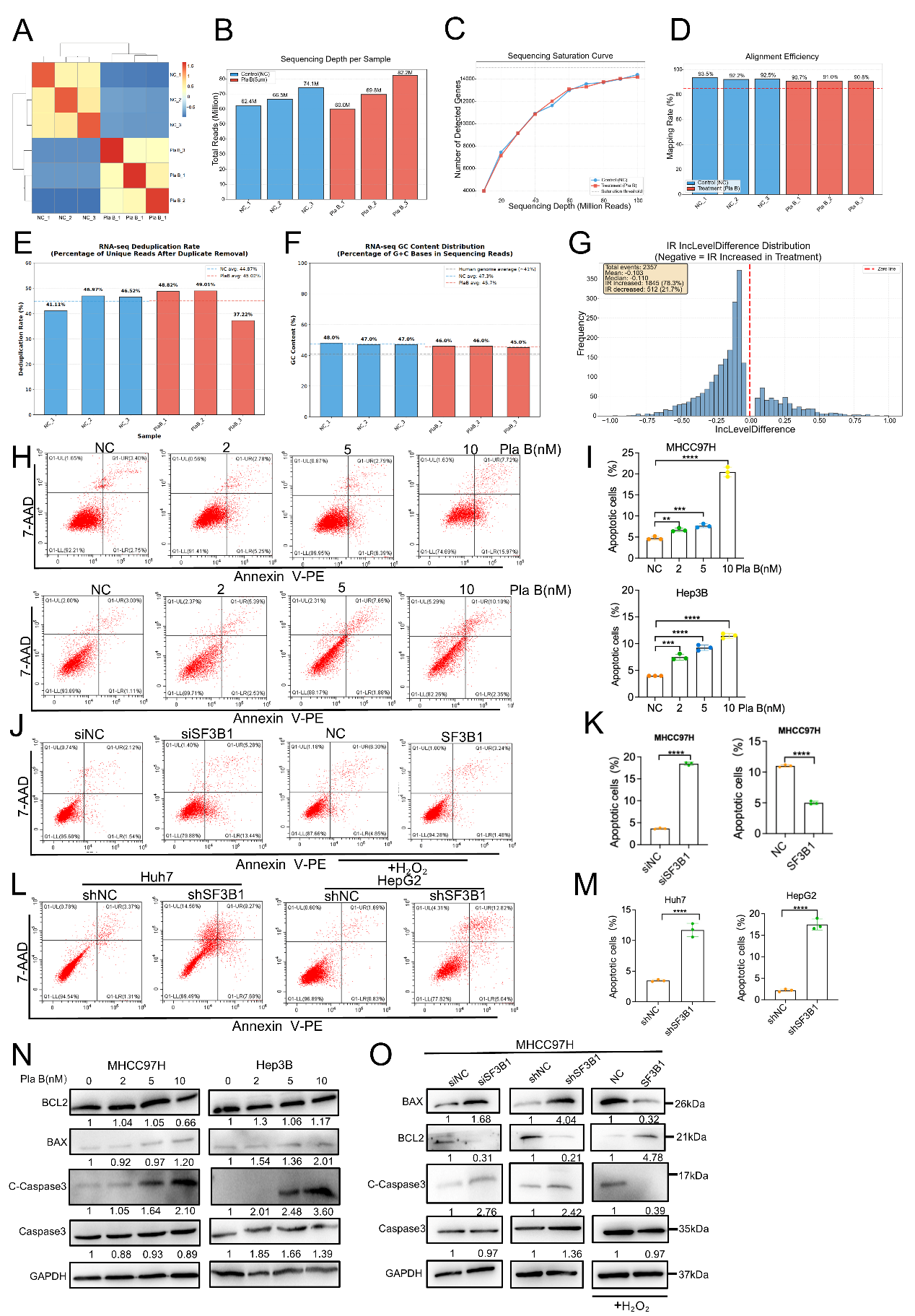


**Figure S3. Knockdown of SF3B1 induces spontaneous apoptosis in HCC cells.** (A) Heatmap of differentially expressed genes identified through RNA-Seq analysis of Huh7 cells treated with DMSO or Pla B (5nM) for 48 h (*n* = 3 each group). (B) The bar chart presents the total reads (a metric of sequencing depth) for each sample included in the RNA-seq analysis of Huh7 cells treated with DMSO or Pla B (5nM) for 48 h (*n* = 3 each group). (C) Sequencing saturation curve showing the number of genes detected in RNA-seq of Huh7 cells treated with DMSO or Pla B (5nM) for 48 h (*n* = 3 each group). (D) Bar chart depicting the sequencing read alignment efficiency (mapping rate ≥ 90%) for each sample in the RNA-seq analysis of Huh7 cells treated with DMSO or Pla B (5nM) for 48 h (*n* = 3 each group). (E) Bar chart depicting the sequencing deduplication rates for each sample in the RNA-seq analysis of Huh7 cells treated with DMSO or Pla B (5nM) for 48 h (*n* = 3 each group). (F) Bar chart depicting the sequencing GC content distribution for each sample in the RNA-seq analysis of Huh7 cells treated with DMSO or Pla B (5nM) for 48 h (*n* = 3 each group). (G) Calculated distribution of retained introns demonstrating enhanced IR. (H-I) Apoptotic cells were detected by flow cytometry after staining with Annexin V-PE/7-AAD in HCC cells (MHCC97Hand Hep3B) after 48 h treatment with different concentrations of Pla B (n = 3 biologically independent experiments). Apoptotic cells percentage included early and late apoptotic cells. (J-K) Apoptotic cell detection via flow cytometry in MHCC97H cell following 48 h treatment with SF3B1 siRNA and SF3B1 overexpression constructs; cells were stained with Annexin V-PE/7-AAD (n = 3 biologically independent experiments). Apoptotic cells percentage included early and late apoptotic cells. (L-M) Apoptotic cell detection via flow cytometry in Huh7 and HepG2 cell following 48 h treatment with shSF3B1 constructs; cells were stained with Annexin V-PE/7-AAD (n = 3 biologically independent experiments). Apoptotic cells percentage included early and late apoptotic cells. (N) Western blot analysis of apoptosis markers after 48 h treatment with different concentrations of Pla B (2nM, 5nM, and 10 nM) and DMSO (control) in HCC cells (MHCC97H and Hep3B). (O) Western blot analysis of apoptosis markers after 48 h treatment with SF3B1 siRNA, SF3B1 shRNA, or SF3B1 overexpression constructs in MHCC97H cell; cells were stained with Annexin V-PE/7-AAD. All data in the figures (I, K, M) were presented as mean ± SD. *P*-values were determined by two-tailed unpaired t-tests. **p* < 0.05, ***p* < 0.01, ****p* < 0.001.


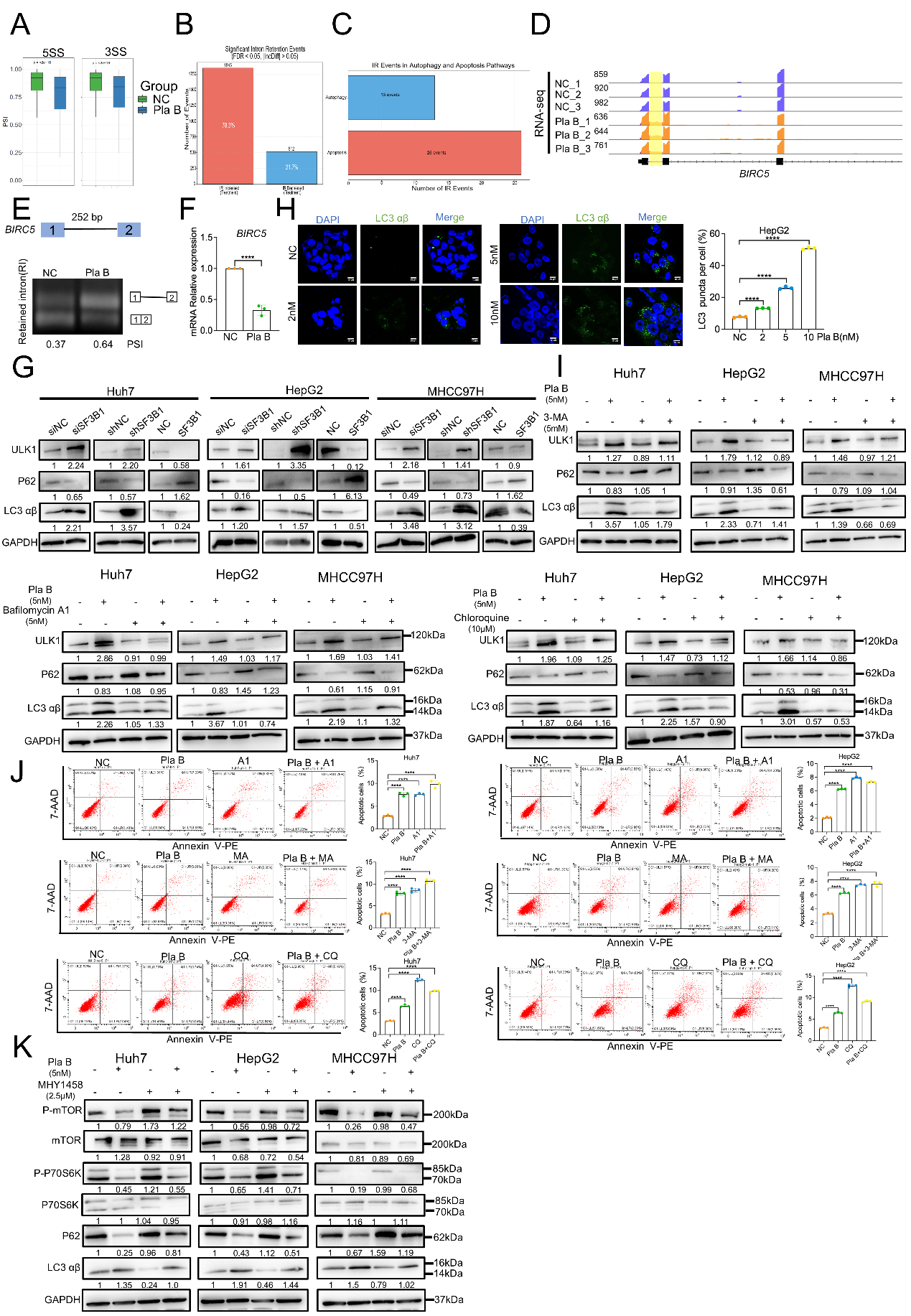


**Figure S4. Pla B decreases the splicing efficiency of HCC cells and promotes autophagy in these cells.** (A) Landscape of splicing efficiency inferred in the RNA-seq analysis of Huh7 cells treated with DMSO or Pla B (5nM) for 48 h (*n* = 3 each group). (B) The bar chart shows the increased and decreased IR events in the autophagy and apoptosis pathways. (C) Events of IR in the autophagy and apoptosis pathways. (**D**)  The AS pattern and SF3B1 direct binding sites in the *BIRC5* pre-mRNA were visualized with IGV using the RNA-seq data in Huh7 cells treated with DMSO or Pla B (5nM) for 48 h (*n* = 3 each group). The yellow region highlights the AS region. (**E**) Semiquantitative RT-PCR was performed to validate AS events of *BIRC5* in Huh7 cells treated with DMSO or Pla B (5nM) for 48 h (*n* = 3 each group). Percent spliced in (PSI) was quantified (*n* = 3). (**F**) RT-qPCR analysis of *BIRC5* expression in Huh7 cells treated with DMSO or Pla B (5nM) for 48 h (*n* = 3 each group). (**G**) Immunoblotting analysis of autophagy markers after 48 h treatment with SF3B1 siRNA, SF3B1 shRNA, or SF3B1 overexpression constructs in HCC cells (Huh7, HepG2 and MHCC97H). (**H**) Immunofluorescence staining of LC3 in control and pladienolide B-treated (48 h) HepG2 cells, with puncta quantification (mean ± SD; n = 3). (I)Western blot analysis of autophagy-related protein expression in HCC cells (Huh7, HepG2 and MHCC97H) treated with Pla B (5nM) alone or in combination with three autophagy inhibitors (3-MA, bafilomycin A1, and chloroquine) for 48 h. (**I**)Western blot analysis of autophagy-related protein expression in HCC cells (Huh7, HepG2 and MHCC97H) treated with Pla B (5nM) alone or in combination with three autophagy inhibitors (3-MA 5 mM, bafilomycin A1 5nM, and chloroquine 10 μM) for 48 h. (**J**) Apoptotic cell detection via flow cytometry in HCC cells(Huh7, HepG2 and MHCC97H) with Pla B (5nM) alone or in combination with three autophagy inhibitors (3-MA, bafilomycin A1, and chloroquine) for 48 h.; cells were stained with Annexin V-PE/7-AAD (n = 3 biologically independent experiments). (**K**)Western blot analysis of mTOR and autophagy-related protein expression in HCC cells (Huh7, HepG2 and MHCC97H) treated with Pla B (5nM) alone or in combination with activation of mTORC1 (MHY1458) for 48 h. All data in the figures (A, F, H, J) were presented as mean ± SD. *P*-values were determined by two-tailed unpaired t-tests. **p* < 0.05, ***p* < 0.01, ****p* < 0.001.


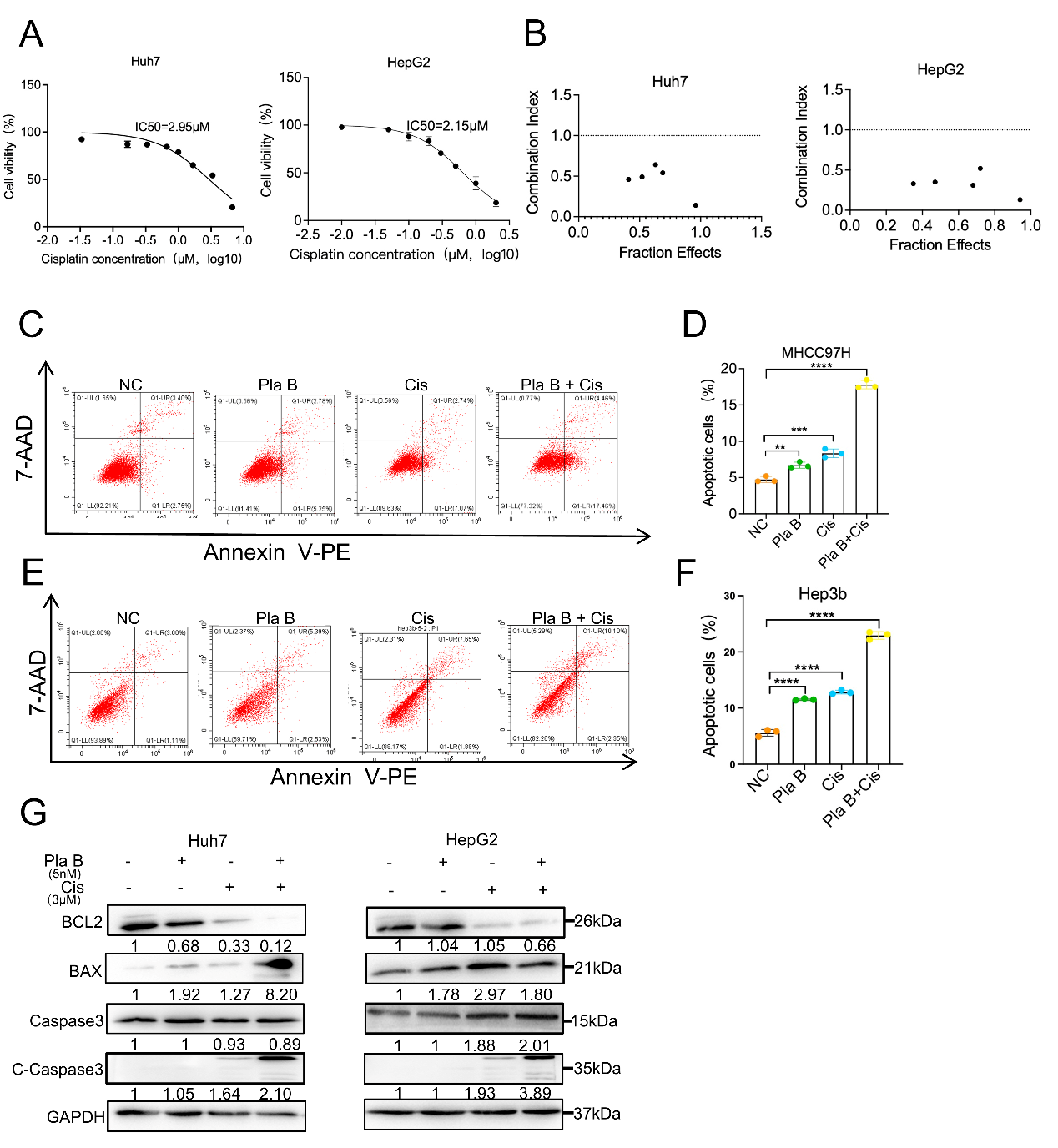


**Figure S5. The combination of Pla B and cisplatin promotes the apoptosis of HCC cells (Huh7 and HepG2).** (A) The MTT cell proliferation assay was employed to evaluate the proliferation of HCC cells (Huh7 and HepG2) treated with cisplatin for 48 hours, and their IC50 values were determined. (B) Combination Index analysis for the combination of Pla B and Cis of HCC cells (Huh7 and HepG2). CI was calculated from Chou-Talalay. (E-F) Cytometric analysis of Hep3B and MHCC97H cells after 48 hours of combined treatment with Pla B (5nM), or cisplatin(3μM), or both drugs. (G) Western blot analysis of apoptosis markers after 48-hour treatment with Pla B (5nM), or cisplatin(3μM), or both drugs. All data in the figures (D and F) were presented as mean ± SD. P-values were determined by two-tailed unpaired t-tests. **p* < 0.05, ***p* < 0.01, ****p* < 0.001.


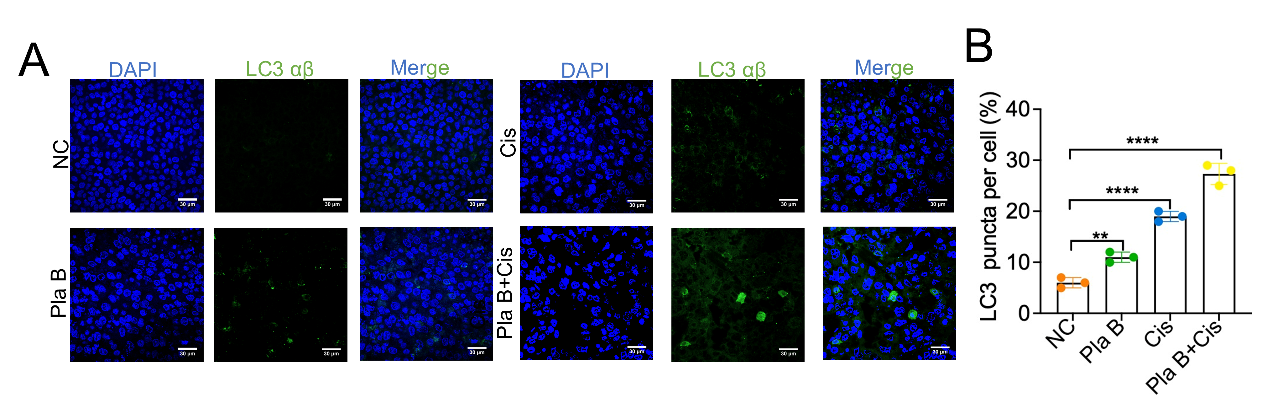


**Figure S6. The combination of PlaB and cisplatin promotes autophagy in HCC.** (A-B) LC3αβ immunofluorescence staining of tumors in the control group and groups treated with either Pla B (5nM), or cisplatin(3μM), or combination therapy. Number of foci counted (mean ± standard deviation; n=3). All data in the figures (B) were presented as mean ± SD. P-values were determined by two-tailed unpaired t-tests. **p* < 0.05, ***p* < 0.01, ****p* < 0.001.


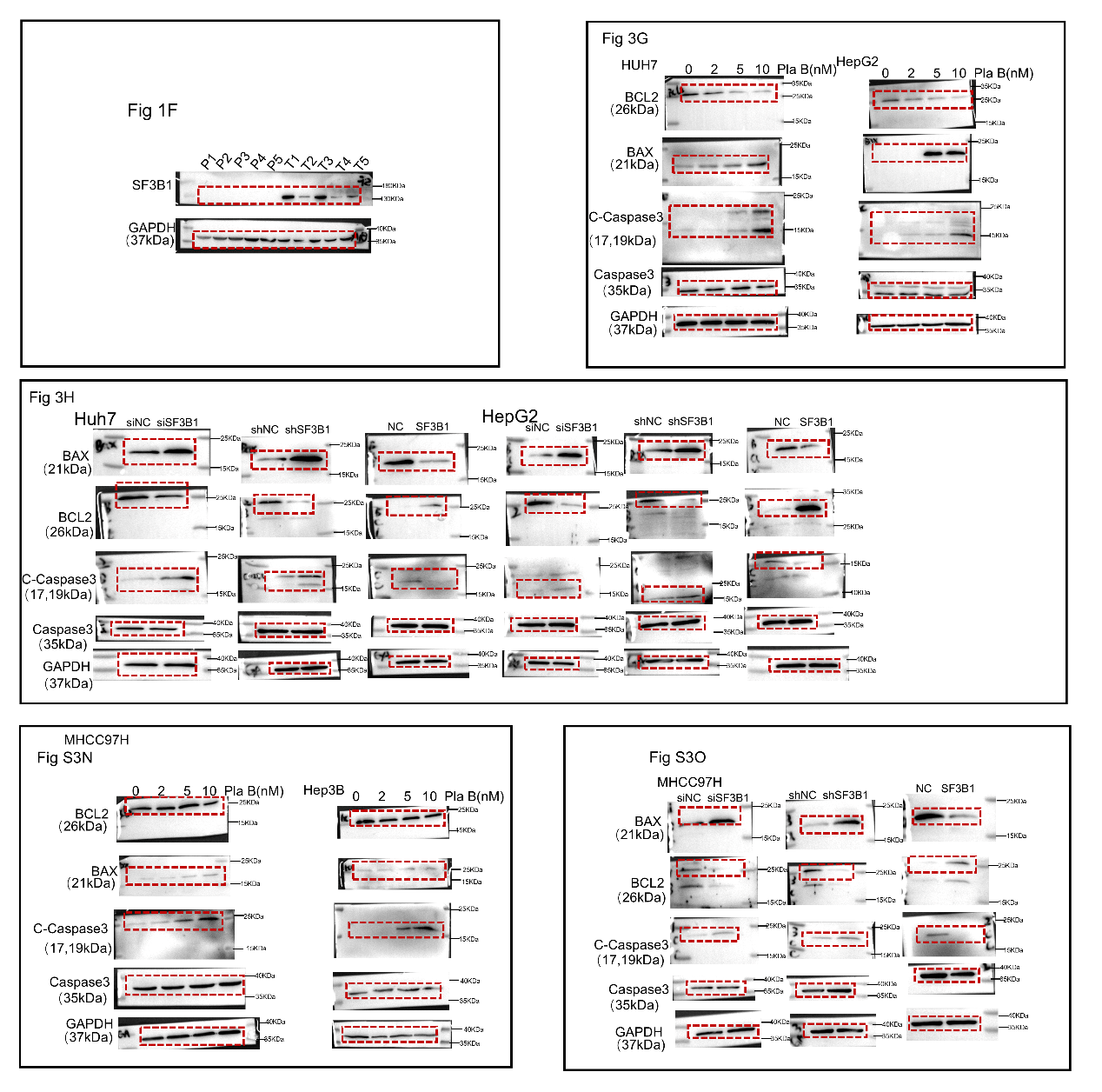


**Figure S7. Full and uncropped western blot images.
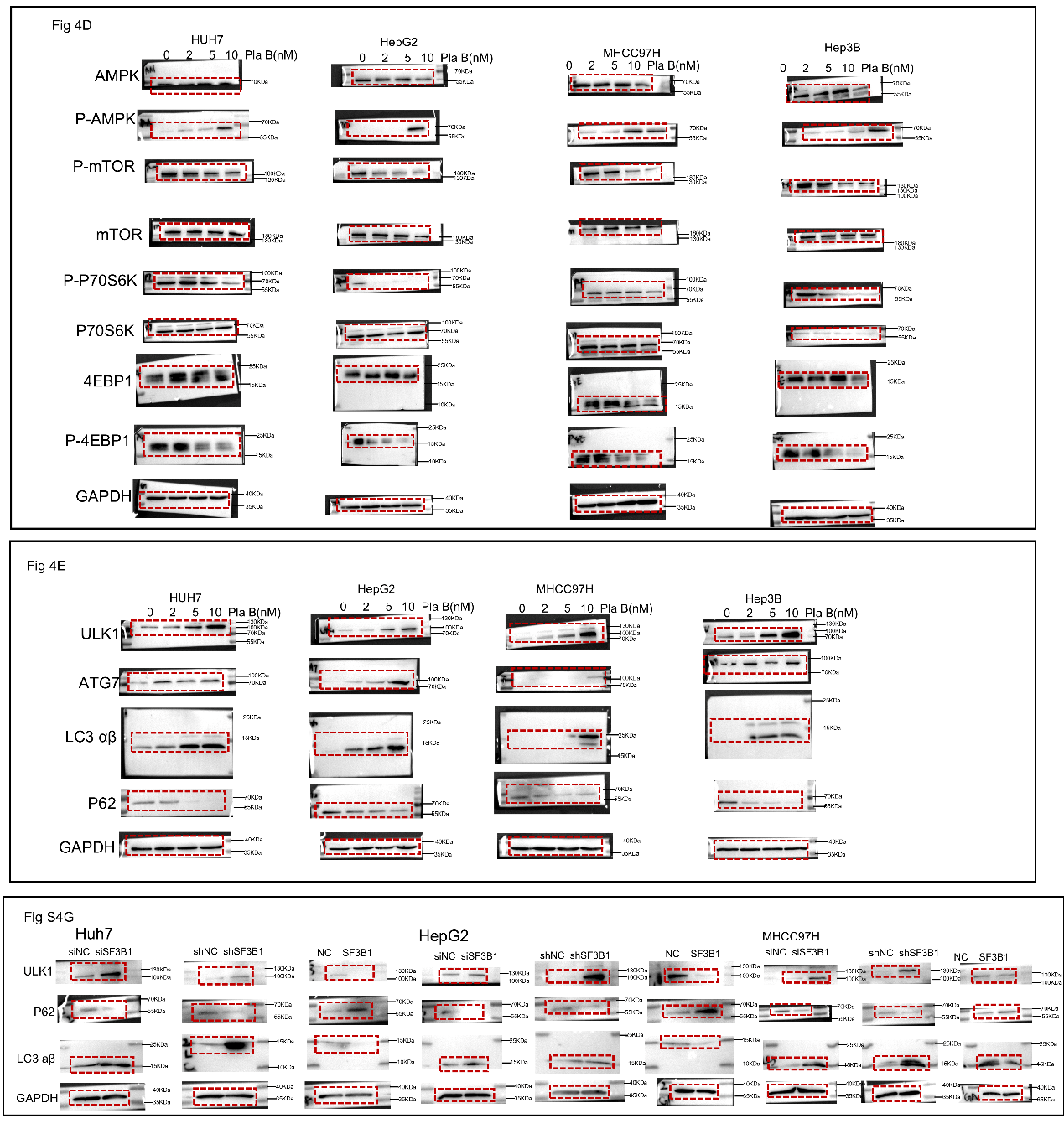
**

**Figure S8. Full and uncropped western blots images.**


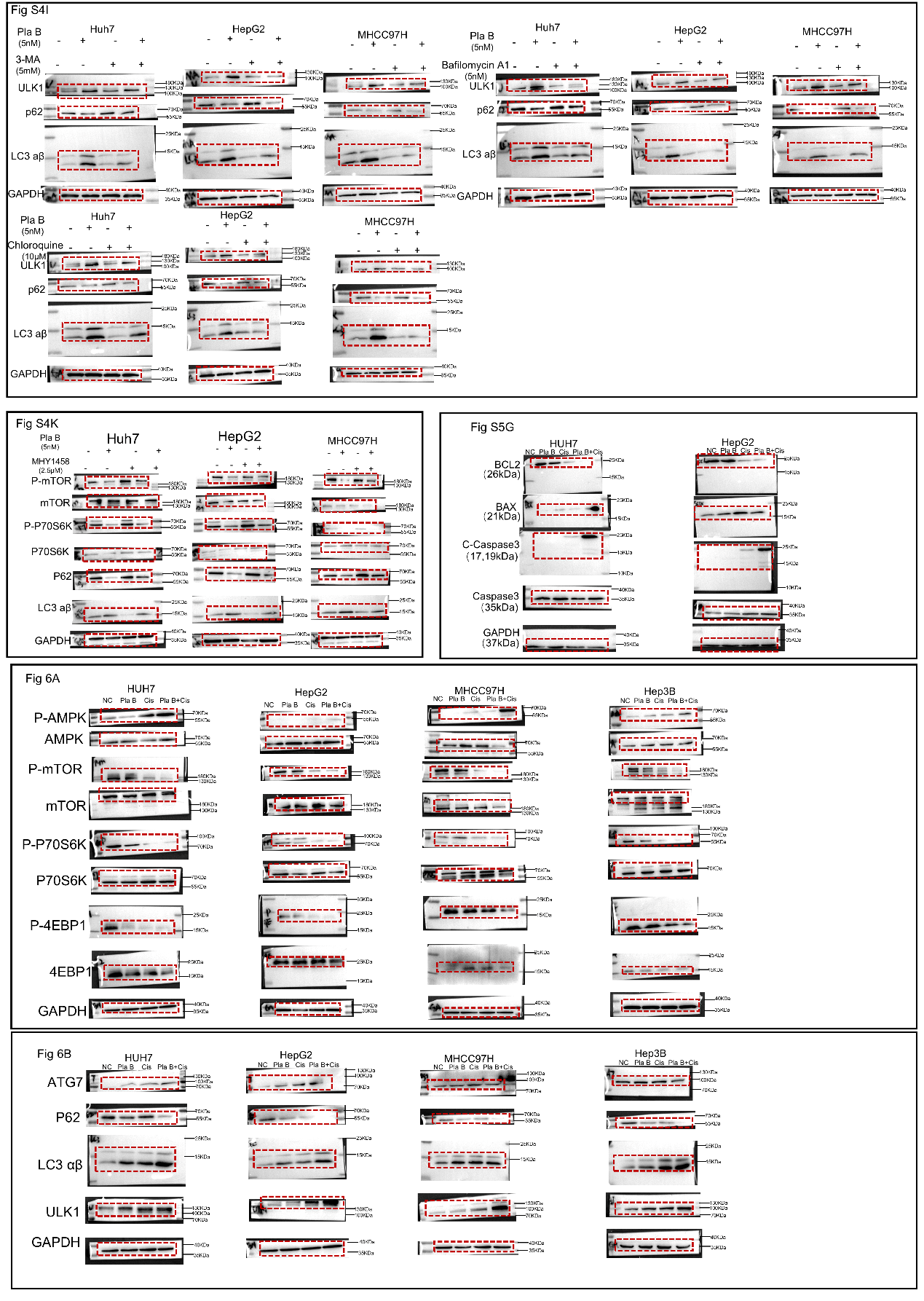


**Figure S9.** **Full and uncropped western blots images.**
